# Vitamins D, A and E, and Beta-Carotene in Adherent and Non-Adherent Individuals with Phenylketonuria: Cross-Sectional Study, Systematic Review and Meta-Analysis

**DOI:** 10.3390/nu17243932

**Published:** 2025-12-16

**Authors:** Kamila Bokayeva, Małgorzata Jamka, Łukasz Kałużny, Monika Duś-Żuchowska, Natalia Wichłacz-Trojanowska, Renata Mozrzymas, Agnieszka Chrobot, Dariusz Walkowiak, Olga Ļubina, Ilya Rabkevich, Szymon Kurek, Anna Miśkiewicz-Chotnicka, Gulnara Sultanova, Karl-Heinz Herzig, Madara Auzenbaha, Jarosław Walkowiak

**Affiliations:** 1 Poznan University of Medical Sciences, Department of Pediatric Gastroenterology and Metabolic Diseases, Szpitalna Str. 27/33, 60-572 Poznań, Poland; kamila.bokayeva@gmail.com (K.B.); mjamka@ump.edu.pl (M.J.); lkaluzny@ump.edu.pl (Ł.K.); mduszuchowska@ump.edu.pl (M.D.-Ż.); natalia.wichlacztrojanowska@ump.edu.pl (N.W.-T.); skurek@ump.edu.pl (S.K.); chotnicka@ump.edu.pl (A.M.-C.); kherzig@ump.edu.pl (K.-H.H.); 2 Poznan University of Medical Sciences, Doctoral School, Bukowska Str. 70, 60-812 Poznań, Poland; 3 Research and Development Center, Department of Pediatrics, Regional Specialist Hospital, Kamienskiego Str. 73a, 51-124 Wrocław, Poland; mozrzymas@wssk.wroc.pl; 4 Metabolic Outpatient Clinic, Voievodship Children Hospital, Chodkiewicza Str. 44, 85-667 Bydgoszcz, Poland; aga.chrobot@wp.pl; 5 Poznan University of Medical Sciences, Department of Organization and Management in Health Care, Marii Magdaleny Str. 14, 61-786 Poznań, Poland; 6 Clinic of Medical Genetics and Prenatal Diagnostics, Children’s Clinical University Hospital, Vienības Gatve Str. 45, 1004 Riga, Latvia; olga.lubina@bkus.lv (O.Ļ.); illia.rabkevich@gmail.com (I.R.); madara.auzenbaha@bkus.lv (M.A.); 7 School of Dentistry, Pharmacy, Nursing, Public Health and Preventive Medicine, West Kazakhstan Marat Ospanov Medical University, Maresyev Str. 68, Aktobe 030019, Kazakhstan; stomfak.zkgmu@mail.ru; 8 Research Unit of Biomedicine and Internal Medicine, Faculty of Medicine, University of Oulu, Aapistie Str. 5, 90220 Oulu, Finland; 9 Biocenter Oulu, University of Oulu, Aapistie Str. 5, 90220 Oulu, Finland; 10 Medical Research Center, Oulu University Hospital, Aapistie Str. 5, 90220 Oulu, Finland

**Keywords:** PKU, inborn errors of metabolism, adherence, vitamins, nutrition, metabolism, diet therapy

## Abstract

**Background/Objectives:** The impact of dietary adherence and formula intake regularity on fat-soluble vitamin status in phenylketonuria (PKU) is uncertain. This study assessed whether vitamin A, D, E, and beta-carotene levels differ by dietary adherence and regularity of Phe-free formula intake. **Methods:** A cross-sectional study included 98 individuals (age 6–41 years) with vitamin D measurements. In a subgroup of 68 patients, vitamin A, vitamin E, and beta-carotene levels were determined. Vitamin levels were compared between adherent and non-adherent groups and between participants with regular vs. irregular formula intake. A subsequent systematic review and meta-analysis of six studies (from PubMed, Scopus, Web of Science, and Cochrane; searched in August 2025) pooled standardised mean differences (SMDs) using fixed-effects and random-effects models. **Results:** The cross-sectional results showed higher vitamin D in adherent (35.60 [30.39–41.65] vs. 32.90 [26.50–40.00] ng/mL, *p* = 0.034) and regular formula consumers (35.97 [30.03–42.28] vs. 30.20 [26.08–35.06] ng/mL, *p* = 0.002). Beta-carotene was elevated with regular intake (74.40 [56.70–98.45] vs. 53.20 [34.10–68.60] ng/mL, *p* = 0.003). Meta-analysis confirmed higher vitamin D in adherent individuals (fixed-effects model, SMD = 0.290, 95% CI: 0.004, 0.576, *p* = 0.047) and regular consumers (fixed-effects model, SMD = 0.750, 95% CI: 0.382, 1.118, *p* < 0.0001). No differences were observed for vitamin E or beta-carotene. **Conclusions:** Adherence to diet and regular formula intake is associated with improved vitamin D status, underscoring the critical role of fortified formulas in PKU management. The very low certainty of evidence necessitates further research, especially for the other fat-soluble vitamins. Nonetheless, clinical practice should emphasise support for adherence and ongoing nutritional monitoring.

## 1. Introduction

Phenylketonuria (PKU) is an autosomal recessive inborn error of metabolism caused by mutations in the gene encoding phenylalanine (Phe) hydroxylase, the hepatic enzyme responsible for converting Phe to tyrosine. In the absence of adequate enzyme activity, Phe accumulates to neurotoxic levels, leading to irreversible intellectual disability and other neurological impairments if untreated [1]. Early diagnosis through newborn screening and immediate initiation of dietary therapy have dramatically improved clinical outcomes. Managing PKU requires lifelong adherence to a Phe-restricted diet and the use of specialised formulas fortified with essential micronutrients [2].

However, commitment to the treatment varies widely among patients of all ages, influencing metabolic control and nutritional status. The complexity of PKU management and social burden can lead to poor adherence—particularly during adolescence and adulthood [3,4]. As individuals transition toward independence, they tend to reduce or discontinue the use of metabolic formulas and increase their intake of Phe-rich foods, resulting in poor metabolic control and potential nutritional imbalances. In this context, attention has increasingly focused on the adequacy of micronutrient intake in PKU. The exclusion of high-protein, nutrient-dense foods, combined with variable adherence to fortified medical formulas, creates a risk of various deficiencies. Fat-soluble vitamins such as A, D, and E, as well as beta-carotene, are of particular interest due to their essential physiological roles and their absorption, which is influenced by dietary composition.

Vitamin A is not a single compound but a group of fat-soluble nutrients that includes two primary forms: preformed vitamin A (retinol, retinal, retinoic acid) and provitamin A carotenoids (beta-carotene, alpha-carotene, and beta-cryptoxanthin) [5,6]. The retinoids are primarily found in animal foods such as beef liver, eggs, and dairy products. In contrast, carotenoids are abundant in plant foods like sweet potato, pumpkin, carrots, apricots, and leafy green vegetables [6,7,8]. Once consumed, beta-carotene is converted in the intestinal mucosa by beta-carotene dioxygenase and subsequently reduced to retinol, the active form of vitamin A in the body [7,9], which modulates immune responses, supports normal reproductive and visual functions, and regulates cellular proliferation and differentiation [7,8,10,11,12,13]. Vitamin A compounds are essential for the proper development and maintenance of vital organs such as the heart, lungs, eyes, and reproductive system [7,8,13,14]. In addition, beta-carotene also functions as an antioxidant, helping to neutralise reactive oxygen species and protect cells from oxidative damage [15]. Reduced plasma beta-carotene in PKU has been reported to correlate negatively with serum Phe concentrations [16], suggesting that poorer metabolic control is associated with lower circulating carotenoid levels. Evaluating beta-carotene alongside other vitamins may provide additional insight into antioxidant capacity and fat-soluble vitamin metabolism in individuals with PKU.

Vitamin E refers to a group of fat-soluble compounds, primarily tocopherols and tocotrienols [17]. Among them, alpha-tocopherol is the most biologically active and the predominant form in human tissues [18]. Rich dietary sources include nuts, seeds, and vegetable oils such as sunflower, safflower, soybean, and wheat germ oil. The vitamin is also found in legumes, butter, tomatoes, and green leafy vegetables [6,19,20]. Vitamin E plays a key role in protecting cell membranes from oxidative damage by inhibiting lipid peroxidation mediated by free radicals [17,18]. Additionally, it stabilises the cell membranes by interacting with destabilising molecules, modulates enzymatic activity, cell signalling, proliferation, and gene expression, and inhibits platelet aggregation [21].

Beyond the restricted intake of many natural vitamin-rich, high-protein sources, some studies have reported evidence of oxidative stress in PKU, including increased markers of lipid, protein, and DNA oxidation, as well as compromised antioxidant defences [22,23,24,25]. Under such conditions, lipophilic antioxidants such as alpha-tocopherol may be utilised more intensively to protect cell membranes from peroxidation, potentially lowering circulating vitamin E levels [26]. Previous studies investigating fat-soluble vitamin status in individuals with PKU have shown variable results. Kose et al. [27] reported higher frequencies of elevated alpha-tocopherol levels among adherent patients than among non-adherent patients (21.9% vs. 8.5%), but no differences in beta-carotene or alpha-tocopherol levels between groups. In contrast, Schulpis et al. [28] found that patients adhering strictly to dietary treatment had significantly higher blood levels of beta-carotene and alpha-tocopherol, along with increased antioxidant status. Colome et al. [22] found suboptimal alpha-tocopherol concentrations in 17.2% of 58 PKU patients. Mikoluc et al. [29] observed that retinol levels remained within normal range despite low intake. However, the latter two studies did not consider adherence.

Vitamin D is obtained from limited dietary sources and from cutaneous synthesis via sunlight exposure, with supplementation being an ordinary and often necessary contributor to status [30]. It is initially inactive and requires two hydroxylation steps to become biologically active. The first occurs in the liver, producing 25-hydroxyvitamin D (25(OH)D), and the second in the kidneys, forming the active hormone 1,25-dihydroxyvitamin D (1,25(OH)_2_D) [31]. Vitamin D is naturally present in fatty fish (e.g., salmon, mackerel), fish liver oils, egg yolks, beef liver, dairy products, and some mushrooms [6,30]. Beyond its well-established role in calcium metabolism and bone health [32], vitamin D also contributes to immune regulation [33], metabolism [34], hematopoietic cell differentiation, and reproductive function [35,36,37]. Emerging evidence links adequate vitamin D levels to reduced risks of certain cancers, including prostate and breast cancer [38,39], and to potential benefits in managing mood disorders such as depression and anxiety [40]. Since individuals with classical PKU may be prone to excess weight due to their specific dietary pattern and use of highly processed low-protein foods, increased adiposity may lower circulating 25(OH)D and contribute to the overall risk of vitamin D deficiency in this population [41]. Vitamin D deficiency is common among individuals with PKU. However, it has been documented that levels are higher than those in the general population [42]. Kose et al. [27] identified 25(OH)D deficiency in 53.6% of 112 patients with PKU. In contrast, Silva et al. [43] reported a lower prevalence of 30.6% in a retrospective study of 90 subjects. Rojas-Agurto et al. [44] observed significantly lower serum vitamin D levels in PKU patients who transitioned from Phe-free protein substitutes to primarily vegan diets compared with those who continued supplementation. In contrast, Hochuli et al. [45] found no differences in 25(OH)D levels between patients with adequate vs. suboptimal amino acid mixture intake.

According to a recent review on nutritional management in PKU [46], in this disorder, strict low-protein diets and reliance on specialised medical foods narrow the range of nutrients reaching the gut, thereby reshaping the gut microbiota and reducing its diversity [47,48,49]. This diet-induced dysbiosis, together with altered production of microbial metabolites, such as short-chain fatty acids, and interactions with nutrient supplements, may have lasting effects on gut health, immune function, and overall metabolism [50,51,52].

Our previous systematic review and meta-analysis [42] indicated that individuals with PKU generally maintain adequate levels of vitamins A, E, and 25(OH)D, comparable to those of healthy controls; however, the impact of dietary adherence and Phe-free formula use on fat-soluble vitamin status remains unclear. To address this gap, the present cross-sectional study evaluates serum vitamins A, D, E and beta-carotene in individuals with PKU, stratified by adherence patterns defined using two criteria: (1) metabolic control (mean annual Phe concentrations) and (2) regularity of prescribed Phe-free formula intake. A systematic review and meta-analysis were conducted to synthesise and assess the available evidence on this topic. We hypothesised that vitamin A, D, E, and beta-carotene levels would not differ significantly between patients with lower versus higher Phe levels, nor between those with regular versus irregular use of Phe-free formula.

## 2. Cross-Sectional Study

### 2.1. Materials and Methods

#### 2.1.1. Study Design and Aim

This study was reported in accordance with the STROBE (Strengthening the Reporting of Observational Studies in Epidemiology) guidelines [53,54], which provide a standardised framework for the transparent and comprehensive reporting of observational research.

This cross-sectional observational study aimed to assess serum levels of vitamins A, D, E, and beta-carotene in individuals with PKU, stratified by annual mean phenylalanine (Phe) levels and prescribed amino acid formula intake.

#### 2.1.2. Sample Size and Inclusion and Exclusion Criteria

The minimum sample size for this study was calculated based on the mean and standard deviation (SD) of vitamin D levels in individuals with PKU who reported regular and irregular formula intake published by Rojas-Agurto et al. [44]. The reported means were 36.97 ± 9.33 nmol/L for the adherent group and 24.3 ± 10.62 nmol/L for the non-adherent group. The difference in means (12.67 nmol/L) and the pooled standard deviation (10.07 nmol/L) were used to estimate the required sample size to detect a significant difference between two independent groups with 80% power and a 95% confidence level, as described by Malone et al. [55]. Using these parameters, the sample size calculation indicated that at least 11 participants per group were needed. To account for potential dropouts or missing data, the target recruitment was increased by 10%, resulting in a minimum total sample size of 26 participants.

Participants eligible for inclusion have been individuals aged 6 years or older with a confirmed diagnosis of classical PKU, defined by a pre-treatment Phe concentration exceeding 1200 μmol/L, identified through a newborn screening programme. All participants have been required to be on continuous dietary treatment and to provide informed consent, either directly or via their parents, for minors.

Exclusion criteria have included acute or chronic medical conditions that could interfere with PKU management or affect the absorption or metabolism of fat-soluble vitamins (A, D, E, and beta-carotene). Individuals undergoing pharmacological treatments such as tetrahydrobiopterin (BH4) or pegvaliase, as well as those currently taking vitamin A or E supplements, have not been considered for the study. Pregnant or breastfeeding participants have also been ineligible for the study.

#### 2.1.3. Participants and Group Division

Patients were recruited from four specialised metabolic centres: the Department of Pediatric Gastroenterology and Metabolic Diseases at Poznan University of Medical Sciences (Poznań, Poland), the Research and Development Center at the Regional Specialist Hospital (Wrocław, Poland), the Voivodship Children’s Hospital (Bydgoszcz, Poland), and the Clinic of Medical Genetics and Prenatal Diagnostics at the Children’s Clinical University Hospital (Riga, Latvia).

Recruitment at the Polish centres occurred from June 2024 to March 2025, and baseline data were collected at enrolment. The Latvian centre recruited participants from January 2024 to December 2024 and specifically assessed vitamin D levels. Informed consent was obtained from all participants, or their legal guardians for individuals under 16 years of age.

Participants were divided into two groups according to two criteria: mean plasma Phe concentration and formula intake. This dual classification was chosen to provide a comprehensive assessment of adherence, capturing both metabolic control, as reflected in Phe levels, and behavioural compliance, as reflected in formula consumption. Adherence by Phe levels was defined using age-specific thresholds consistent with clinical guidelines [1]:

- 120–360 μmol/L (2–6 mg/dL) for children between 6 and 12 years,
- 120–600 μmol/L (2–10 mg/dL) for those aged 12 to 18 years,
- <600 μmol/L (10 mg/dL) for adults.

Patients with mean Phe levels above these cutoffs were considered non-adherent, whereas those at or below the cutoffs were classified as adherent.

Formula intake was categorised as regular when patients consistently consumed the prescribed amount and as irregular when intake was inconsistent or incomplete (i.e., less than two-thirds of the recommended formula), as determined from clinical records and dietary interviews.

#### 2.1.4. Obtained Data

The collected data included birth date, sex, weight, height, and plasma Phe concentrations. In all participants, vitamin A, D, and E, and beta-carotene levels were investigated. BMI was calculated by dividing weight (in kilograms) by height (in metres) squared. For participants aged 18 years or younger, BMI was adjusted using the International Obesity Task Force (IOTF) standards [56]. Mean and median plasma Phe concentrations were calculated, along with the percentages of abnormal values. Mean and median plasma Phe concentrations were retrieved from clinical records covering the preceding 24 months.

#### 2.1.5. Vitamin Assessment

Serum vitamins A, E, and beta-carotene were quantified by high-performance liquid chromatography with UV detectionon a Supelco C18 column using a Hewlett-Packard 1100 Series system (Waldbronn, Germany). Retinol was monitored at 326 nm, alpha-tocopherol at 292 nm, and beta-carotene at 450 nm. Under these conditions, the mean retention times were approximately 3.1 min for vitamin A, 5.5 min for vitamin E, and 15.0 min for beta-carotene. The mobile phase was predominantly methanol (methanol:ethanol mixture), delivered at 0.75–1.4 mL/min, depending on the run conditions. Samples were deproteinised with ethanol and extracted with hexane. This routine method has been described in detail in previous publications from our institution [57,58]. Vitamin D status was assessed by measuring serum 25(OH)D concentrations using an immunoassay method on the Alinity i analyser and electrochemiluminescence immunoassay on a Siemens Atellica IM system equipped with a Cobas e 601 module, performed in commercial laboratories (Diagnostyka S.A., Poznań, Poland & E. Gulbis Laboratory, Riga, Latvia, respectively). Serum vitamin A levels were assessed using a reference range set at 300–750 ng/mL [59,60]. Vitamin D levels were measured, with the reference range provided by Polish guidelines [61] (Table 1). Serum vitamin E levels were evaluated within the reference range of 5.7–19.9 mg/L [62]. The reference interval for serum beta-carotene was 30–910 ng/mL [63,64]. All biochemical measurements were taken from fasting morning blood samples. Vitamin D supplementation status was recorded for all participants.

#### 2.1.6. Intake Assessment

Phe-free formula intake was assessed from patient records and self-report. For each participant, intake of Phe-free formula during the month preceding blood sampling was recorded, and patients or carers documented the number and size of daily doses. In addition, clinical records from the preceding 12 months were reviewed to characterise longer-term formula use and to verify the regularity of reported intake.

#### 2.1.7. Ethics Approval

The study was conducted in accordance with the principles of the Declaration of Helsinki [65] and received approval from the Poznan University of Medical Sciences Ethical Committee (approval number 260/24; approval date: 10 April 2024). All collected data were fully anonymised and securely stored in strict adherence to ethical guidelines and institutional policies.

#### 2.1.8. Statistical Analysis

For the study, the data were summarised using the medians with interquartile ranges (IQRs) and means with SD. Vitamin levels were reported with corresponding 95% confidence intervals (CIs). The Shapiro–Wilk test was applied to assess the normality of the variables. For data that did not follow a normal distribution, the Mann–Whitney U test was used for group comparisons. When data were normally distributed, Levene’s test of homogeneity of variances was conducted to assess whether the variances were equal across groups. If variances were homogeneous, Student’s *t*-test for independent samples was performed; otherwise, Welch’s t-test was used. Pearson’s chi-square test was employed to compare categorical variables across groups. For comparisons with minor expected frequencies, Fisher’s exact test was used. A *p*-value of <0.05 was considered statistically significant. Statistical analyses were carried out using PQStat (PQStat Software, v.1.8.6, Poznan, Poland) and RStudio (Posit Software, v. 2025.05.1 + 513, PBC, Boston, MA, USA) [66].

#### 2.1.9. Bias Control

To minimise selection bias, participants were consecutively recruited from multiple specialised metabolic centres across Poland and Latvia, thereby ensuring a representative sample of individuals with PKU across groups. Standardised inclusion and exclusion criteria were applied uniformly across all sites. Measurement bias was minimised by employing validated instruments and standardised data collection procedures. Laboratory personnel were blinded to participants’ adherence status during sample analysis to reduce detection bias, with group classification performed only after the analysis results were collected. To limit information bias, formula adherence was assessed through clinical records and dietary interviews.

### 2.2. Results

**Figure 1:**
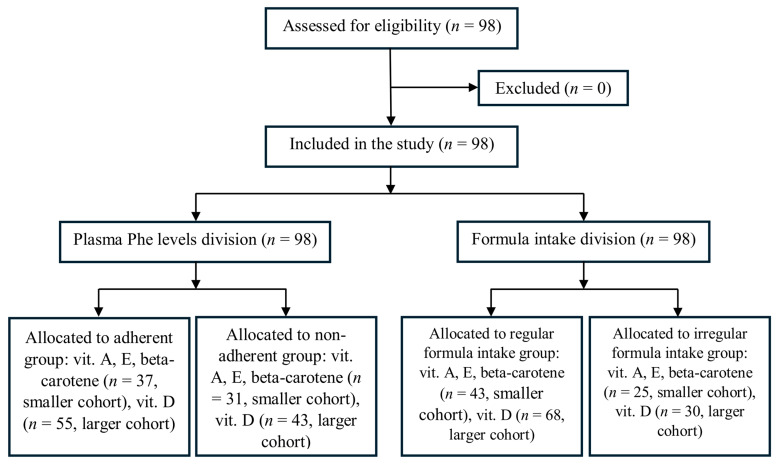
Participant flow diagram.

A total of 98 individuals (cohort L) aged 6 to 41 years were identified as potentially eligible and participated in the study (Figure 1). No participants were withdrawn or excluded. Of these, 68 were recruited from three centres in Poland (cohort S), where complete biochemical analyses, including vitamins A, D, E, and beta-carotene, were performed; 30 participants were recruited from a Latvian centre, where only vitamin D levels were assessed. In total, 14 of the 98 participants (14%) reported using vitamin D supplements.

Table 2 presents serum vitamin concentrations in adherent and non-adherent individuals with PKU, categorised by their annual mean plasma Phe levels. Two partially overlapping cohorts were analysed: a smaller cohort (*n* = 68; 37 adherent, 31 non-adherent) with measurements of vitamins A, E, and beta-carotene (upper part of Table 2), and a larger cohort (*n* = 98; 55 adherent, 43 non-adherent) with vitamin D measurements (lower part of Table 2).

In the smaller cohort, there were no significant differences in age (19 [14–27.80] vs. 19.9 [16.20–28.75] years; *p* = 0.345), sex distribution (female: 62.2% vs. 48.4%, *p* = 0.254), or BMI between adherent and non-adherent groups (21.90 [19.80–24.09] vs. 21.68 [19.77–28.77] kg/m^2^; *p* = 0.161). In the adherent group, 5 (13.2%) participants had elevated serum vitamin A levels, compared to 5 (16.0%) participants in the non-adherent group. No cases of vitamin A deficiency were observed in either group. All participants had vitamin E levels within the reference range. For beta-carotene, the proportion of participants with optimal levels was 86.5% (32 participants) in the adherent group versus 90.3% (28 participants) in the non-adherent group. Deficiencies were documented in 5 (13.5%) and 3 (9.7%) individuals, respectively. The difference was not statistically significant (*p* = 0.719). There were no significant differences in vitamin A, E, or beta-carotene levels between groups (all *p* > 0.05).

In the larger cohort, no statistically significant differences were observed for age (16.6 [12.35–27.40] vs. 19.1 [15.45–26.70] years, *p* = 0.206) or sex distribution (female: 54.5% vs. 48.8%, *p* = 0.575), whereas BMI did not differ between groups (21.52 [18.18–24.10] vs. 21.81 [19.76–27.47], *p* = 0.075). In the adherent group, the majority of individuals had optimal vitamin D levels (*n* = 35, 63.6%), followed by high levels in 8 (14.5%) and suboptimal levels in 12 (21.8%). In the non-adherent group, suboptimal vitamin D levels were observed in 18 (41.9%) individuals, while optimal levels were documented in 22 (51.2%) individuals. Additionally, 2 (4.7%) individuals had high levels, and 1 (2.3%) had a significant vitamin D deficiency. Vitamin D deficiency and suboptimal levels were statistically more frequent (*p* = 0.018), in non-adherent patients. Vitamin D levels were significantly different between adherent and non-adherent groups (35.60 [30.39–41.65] vs. 32.90 [26.50–40.00] ng/mL; *p* = 0.034).

Across both cohorts, mean and median Phe concentrations were significantly higher in non-adherent patients, as was the percentage of abnormal Phe values, clearly indicating better metabolic control among adherent individuals.

Table 3 compares parameters in patients with PKU who received regular versus irregular Phe-free formula intake. As in Table 2, the upper part presents the smaller cohort with vitamins A, E, and beta-carotene measured (*n* = 68; 43 participants with regular intake, 25 participants with irregular intake), and the lower part shows the larger cohort with vitamin D assessed (*n* = 98; 68 participants with regular intake, 30 participants with irregular intake).

In the smaller cohort, regular users were significantly younger (18.4 [13.75–25.55] vs. 24.2 [17.0–29.6] years; *p* = 0.026) and had a lower BMI (21.46 ± 3.64 vs. 25.01 ± 5.35 kg/m^2^; *p* = 0.005) than users with irregular formula intake. Sex distribution did not differ significantly (female: 60.5% vs. 48%, *p* = 0.318). In the regular intake group, elevated serum vitamin A levels were observed in 8 (18.2%) participants, compared to 2 (8.0%) participants in the irregular intake group. 40 (93.0%) participants in the regular intake group had optimal beta-carotene levels, compared with 20 (80%) in the irregular intake group. Deficiency rates did not differ, with 7.0% (3 individuals) in the regular intake group and 20.0% (5 individuals) in the irregular intake group. Although vitamin A and vitamin E levels did not differ significantly between groups (*p* = 0.274 and *p* = 0.559, respectively), beta-carotene concentrations were considerably higher in the regular formula intake group (74.40 [56.70–98.45] vs. 53.20 [34.10–68.60] ng/mL, *p* = 0.003).

In the larger cohort that included 25(OH)D measurements, a similar pattern was seen. Regular users were significantly younger than irregular users (16.65 [12.10–24.53] vs. 22.8 [15.75–29.38] years; *p* = 0.015), and BMI was lower in the regular group (21.16 [17.96–23.44] vs. 24.16 [21.10–29.03] kg/m^2^; *p* = 0.002). Again, no significant difference in sex distribution was found (female: 52.9% vs. 50%, *p* = 0.788). In the regular intake group, most individuals (*n* = 41; 60.3%) had optimal vitamin D levels, with suboptimal levels in 17 (25.0%) and high levels in 10 (14.7%). In the irregular intake group, optimal vitamin D levels were present in 16 (53.3%) individuals, suboptimal levels in 13 (43.3%), and one (3.3%) individual had a significant deficiency. Vitamin D deficiency and suboptimal levels were more frequent among individuals with irregular intake (*p* = 0.034). Vitamin D levels were significantly higher in the regular intake group (35.97 [30.03–42.28] vs. 30.20 [26.08–35.06] ng/mL; *p* = 0.002), further supporting a relationship between regular formula intake and improved micronutrient status.

Patients with irregular intake exhibited markedly elevated mean and median Phe concentrations across both datasets, along with significantly higher proportions of abnormal Phe values, reflecting poorer metabolic control.

## 3. Systematic Review and Meta-Analysis

### 3.1. Materials and Methods

#### 3.1.1. Protocol and Registration

This systematic review and meta-analysis were designed in accordance with established methodological standards, including the Preferred Reporting Items for Systematic Reviews and Meta-Analyses (PRISMA) guidelines [67] and the Cochrane Handbook for Systematic Reviews of Interventions [68].

Before initiating the review, we developed a study protocol specifying all key methodological aspects. To ensure transparency and reduce potential biases, the protocol was registered with International Prospective Register of Systematic Reviews (PROSPERO), where it is publicly available under registration number CRD420251128538 [69].

#### 3.1.2. Inclusion and Exclusion Criteria

Study Design and Language: We included only human studies published in English in peer-reviewed scientific journals. Eligible studies had to use a cross-sectional design and report data comparing vitamin A, D, E, or beta-carotene levels between adherent and non-adherent individuals with PKU.

Population: Included participants were individuals diagnosed with classical PKU during the neonatal period and managed with early initiation of dietary treatment, specifically a Phe-restricted diet.

Data Requirements: Studies had to report detailed, extractable information, including participant numbers, demographic data, and specific values for vitamin A, D, E, and beta-carotene for both groups being compared.

Exclusion Criteria:

- Mild hyperphenylalaninemia;
- Pregnant or lactating women;
- Individuals receiving supplementation specifically with vitamins A, E, or beta-carotene beyond the usual dietary management;
- Patients undergoing treatment with BH4 or pegvaliase;
- Conference abstracts, abstract-only publications, and studies lacking complete data or vitamin measurements.

Two classification methods were applied: mean plasma Phe level cut-offs and regularity of metabolic formula intake.

Division by mean Phe levels: Adherence to dietary management was defined based on reported mean plasma Phe levels. It was classified according to the age-specific thresholds established in European guidelines [1]:

- Children between 0 and 12 years: mean Phe 120–360 μmol/L (2–6 mg/dL);
- Individuals aged 12 to 18 years: mean Phe 120–600 μmol/L (2–10 mg/dL);
- Individuals older than 18 years: <600 μmol/L (10 mg/dL).

Participants exceeding the respective thresholds were considered non-adherent.

Division by formula intake regularity:

- Regular intake: individuals consuming the recommended amounts of Phe-free metabolic formula consistently, as per dietary prescription;
- Irregular intake: individuals with inconsistent or insufficient intake of metabolic formula, based on study definitions or dietary records.

Studies were included only if they provided stratification by adherence or formula consumption or if adherence status could be calculated from reported mean plasma Phe levels.

#### 3.1.3. Data Collection Process, Extraction and Analysis

Two independent reviewers (K.B. and M.J.) conducted the literature screening in three sequential phases: initial title screening, abstract screening, and full-text review. At each stage, studies that failed to meet the inclusion criteria or were duplicates were systematically excluded. To ensure comprehensive coverage, any study flagged as potentially relevant by either reviewer advanced to subsequent evaluation rounds. Discrepancies in study eligibility judgements were resolved through consultation with a senior researcher (J.W.), who served as an arbitrator when consensus could not be reached. When critical data elements were incomplete or unclear, we contacted the corresponding authors for clarification or additional information.

All references were systematically organised using the reference management tool Zotero (version 7.0.22, https://www.zotero.org/, accessed on 14 June 2025). For the systematic review, we established a minimum threshold of two independent studies reporting on each vitamin of interest. Where data permitted, we performed meta-analyses to quantitatively assess differences in vitamin concentrations between adherent and non-adherent PKU populations.

#### 3.1.4. Data Item

The extracted information from each included article consisted of the following:

1. General information: the title of the article, journal name, primary author, and publication year.
2. Study characteristics: the study name and design, country (region), and sample size (total number of subjects and the number in each group who were included and completed the study).
3. Study population characteristics: age, sex, BMI (kg/m^2^).
4. Description of dietary treatment: natural protein intake (g/day), protein substitute intake (g/day), total protein intake (g/day), Phe intake (mg/d), annual mean/median Phe levels (μmol/L), follow-up (yes or no), treatment adherence (yes or no), Phe levels (μmol/L), tyrosine levels (μmol/L).
5. Primary outcomes: serum or plasma levels of vitamins A (ng/mL), D (ng/mL), E (µg/mL), and beta-carotene (ng/mL).

#### 3.1.5. Information Sources and Search Strategy

We conducted an exhaustive search across four major biomedical databases (PubMed/Medline, Scopus, Web of Science, and Cochrane Library) in August 2025. Our search strategy was designed to capture all available evidence on blood concentrations of vitamins A, D, E, and beta-carotene in patients with PKU, with particular focus on differences between dietary adherent and non-adherent populations and between patients with regular and irregular formula intake.

We considered both experimental studies (including randomised and non-randomised controlled trials) and observational studies (cross-sectional and case–control designs) eligible for inclusion. To ensure historical completeness, we applied no publication date restrictions, allowing our review to encompass the full timeline of relevant research.

To mitigate potential database retrieval limitations, we implemented two supplemental approaches:

- Hand-searching of reference lists from included studies;
- Review of citations in relevant meta-analyses and systematic reviews.

The search strategy combined terms related to PKU and micronutrient status, including “PKU”, “phenylketonuria”, “vitamin”, “diet”, “nutrition”, and their synonyms or related keywords.

We optimised search sensitivity by:

- Employing controlled vocabulary Medical Subject Headings (MeSH);
- Consulting existing systematic reviews for additional relevant terms;
- Utilising database-specific search syntax to account for platform differences.

Detailed search strategy was following:

Cochrane: “phenylketonuria” OR “phenylalanine hydroxylase deficiency” OR “phenylalanine hydroxylase deficient” OR “PKU” OR “hyperphenylalaninaemia” OR “BH4 deficiency” OR “BH4 deficient” OR “tetrahydrobiopterin deficiency” OR “tetrahy-drobiopterin deficient” OR “PAH deficiency” OR “PAH deficient” OR “phenylketonuric” OR “hyperphenylalaninaemic” in Title Abstract Keyword—(August 2025).

PubMed: (“phenylketonuria” OR “phenylalanine hydroxylase deficiency” OR “phenylalanine hydroxylase deficient” OR “PKU” OR “hyperphenylalaninaemia” OR “BH4 deficiency” OR “BH4 deficient” OR “tetrahydrobiopterin deficiency” OR “tetrahydrobiopterin deficient” OR “PAH deficiency” OR “PAH deficient” OR “phenylketonuric” OR “hyperphenylalaninaemic” [MeSH Terms]) AND (“dietary” OR “supplement” OR “supplementations” OR “supplementation” OR “nutritional” OR “nutrition” OR “diet” OR “diets” OR “vitamin” OR “vitamins” OR “vitaminization” OR “vitaminisation” OR “nutrient” OR “nutrients” OR “micronutrient” OR “micronutrients” OR “fat-soluble” OR “fat soluble” OR “calciferol” OR “cholecalciferol” OR “colecalciferol” OR “ergocalciferol” OR “dihydroxycholecalciferol” OR “1,25(OH)2D” OR “hydroxyvitamin” OR “25(OH)D” OR “hydroxyergocalciferol” OR “tocopherol” OR “tocopherols” OR “antioxidant” OR “antioxidants” OR “carotenoid” OR “carotenoids” OR “retinol” OR “retinal” OR “retinoic” OR “retinyl” OR “carotene” OR “provitamin” OR “provitamins” [MeSH Terms])—(August 2025).

Scopus: (TITLE-ABS-KEY (“phenylketonuria” OR “phenylalanine hydroxylase deficiency” OR “phenylalanine hydroxylase deficient” OR “PKU” OR “hyperphenylalaninaemia” OR “BH4 deficiency” OR “BH4 deficient” OR “tetrahydrobiopterin deficiency” OR “tetrahydrobiopterin deficient” OR “PAH deficiency” OR “PAH deficient” OR “phenylketonuric” OR “hyperphenylalaninaemic”) AND TITLE-ABS-KEY (“dietary” OR “supplement” OR “supplementations” OR “supplementation” OR “nutritional” OR “nutrition” OR “diet” OR “diets” OR “vitamin” OR “vitamins” OR “vitaminization” OR “vitaminisation” OR “nutrient” OR “nutrients” OR “micronutrient” OR “micronutrients” OR “fat-soluble” OR “fat soluble” OR “calciferol” OR “cholecalciferol” OR “colecalciferol” OR “ergocalciferol” OR “dihydroxycholecalciferol” OR “1,25(OH)2D” OR “hydroxyvitamin” OR “25(OH)D” OR “hydroxyergocalciferol” OR “tocopherol” OR “tocopherols” OR “antioxidant” OR “antioxidants” OR “carotenoid” OR “carotenoids” OR “retinol” OR “retinal” OR “retinoic” OR “retinyl” OR “carotene” OR “provitamin” OR “provitamins”)—(August 2025).

Web of Science: “phenylketonuria” OR “phenylalanine hydroxylase deficiency” OR “phenylalanine hydroxylase deficient” OR “PKU” OR “hyperphenylalaninemia” OR “BH4 deficiency” OR “BH4 deficient” OR “tetrahydrobiopterin deficiency” OR “tetrahy-drobiopterin deficient” OR “PAH deficiency” OR “PAH deficient” OR “phenylketonuric” OR “hyperphenylalaninaemia” (Topic) AND “dietary” OR “supplement” OR “supplementations” OR “supplementation” OR “nutritional” OR “nutrition” OR “diet” OR “diets” OR “vitamin” OR “vitamins” OR “vitaminization” OR “vitaminisation” OR “nutrient” OR “nutrients” OR “micronutrient” OR “micronutrients” OR “fat-soluble” OR “fat soluble” OR “calciferol” OR “cholecalciferol” OR “colecalciferol” OR “ergocalciferol” OR “dihydroxycholecalciferol” OR “1,25(OH)2D” OR “hydroxyvitamin” OR “25(OH)D” OR “hydroxyergocalciferol” OR “tocopherol” OR “tocopherols” OR “antioxidant” OR “antioxidants” OR “carotenoid” OR “carotenoids” OR “retinol” OR “retinal” OR “retinoic” OR “retinyl” OR “carotene” OR “provitamin” OR “provitamins” (Topic)—(August 2025).

#### 3.1.6. Risk of Bias of Individual Studies

Given the inclusion of non-randomised studies in our meta-analysis, we employed the Newcastle-Ottawa Scale (NOS) [70], a validated tool for assessing methodological quality in observational research. For cross-sectional studies, we applied the modified NOS version developed by Modesti et al. [71], which includes an additional evaluation criterion better suited to this study design.

The NOS examines three domains of study quality:

- Selection (maximum five stars): Evaluates how well the study sample reflects the target population, whether the sample size is adequately justified, how the study handles non-respondents, and the reliability of methods used to measure exposure (e.g., vitamin levels);
- Comparability (maximum two stars): Evaluates whether key confounding factors are controlled for in the study design or analysis;
- Outcome (maximum three stars): Examines the objectivity of outcome assessment and the appropriateness and clarity of statistical analysis.

The methodological quality of included studies was assessed using a standardised scoring system, with cross-sectional studies scored on a 10-point scale. Higher points corresponded to higher study quality and lower risk of bias. Studies were assessed as having low risk of bias (scores ≥ 7), moderate risk (scores 5–6), or high risk (scores ≤ 4).

An independent evaluation was conducted by two reviewers (K.B. and M.J.). When disagreements arose, they formally discussed the discrepancies and reached a consensus, thereby ensuring uniform application of the evaluation criteria across all studies.

#### 3.1.7. Certainty of Evidence Assessment

We used the Grading of Recommendations, Assessment, Development, and Evaluation (GRADE) framework [72] to assess the quality of evidence in our data. The assessment was carried out by two independent researchers (K.B. & M.J.), who resolved any discrepancies through discussion.

#### 3.1.8. Data Synthesis and Analysis

We conducted all meta-analyses using Comprehensive Meta-Analysis Software (version 3.0, Biostat, Inc., Englewood, CO, USA), evaluating each vitamin separately when at least two studies provided comparable data. We calculated effect sizes using the mean and SD of vitamin concentrations from each study. This approach allowed us to compare group differences in a standardised, quantitative manner. When studies reported results using median and range, we attempted to contact the original authors to obtain the necessary data. For one included study [73], our team performed calculations using the raw data provided by the authors. To harmonise results across studies using different measurement scales, we calculated standardised mean differences (SMDs) with 95% CIs. This approach standardised all vitamin concentration measurements by expressing differences in standard deviation units, thereby improving clinical interpretability. SMDs were derived by dividing the difference in group means by the pooled SD of the outcome values. To account for methodological variability across studies, heterogeneity was assessed using two statistical approaches: the Cochran Q statistic (*p* < 0.1 indicating significant heterogeneity) and the *I*^2^ statistic. The *I*^2^ statistic was interpreted as:

- Low variation: <25%;
- Moderate variation: 25–50%;
- High variation: >75%.

We adapted our approach based on these findings—employing fixed-effect models when studies showed remarkable consistency, but switching to random-effects models when moderate or high heterogeneity emerged. Forest plots were generated to visually display the effect sizes and confidence intervals for each study. To assess the robustness of the results, sensitivity analyses were conducted by sequentially removing individual studies and recalculating the pooled effect. In addition, studies at high risk of bias were excluded from separate analyses to assess their influence on the overall outcomes. Publication bias was examined using Begg’s and Egger’s tests, and a cumulative meta-analysis was also performed to observe how the evidence evolved. A *p*-value < 0.05 was considered statistically significant.

### 3.2. Results

#### 3.2.1. Search Results

**Figure 2:**
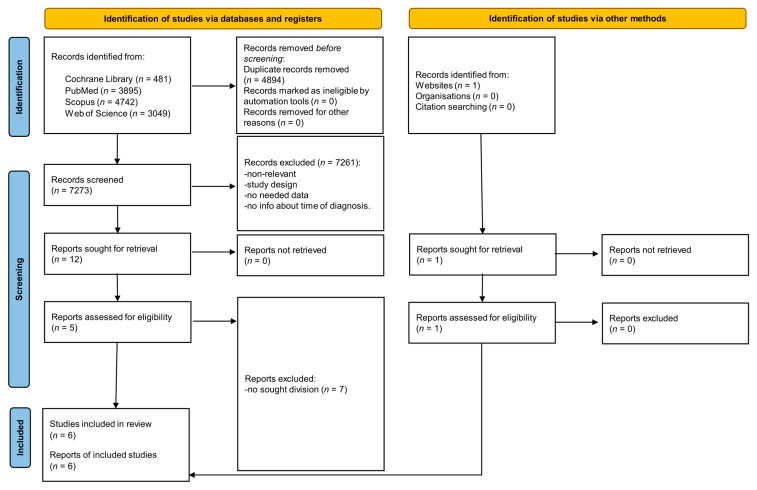
PRISMA 2020 flow diagram.

From the initial 12,167 articles, 4894 duplicates were excluded. After screening the titles and abstracts, 13 relevant articles were identified, of which 6 met the inclusion criteria (Figure 2).

#### 3.2.2. Study Characteristics

The studies included in the analysis were published between 2003 [28] and 2025 [74] (+present study), and their characteristics are summarised in Table 4 and Table 5. Earlier research was conducted in Greece [28], followed by studies in Spain [73], Switzerland [45], Turkey [27,74], and Chile [44], with the present investigation undertaken in Poland. All studies were cross-sectional. The number of participants in the groups varied from 5 [45] to 71 [27]. Two studies included participants under 18 years of age [28,74]; two studies focused on adults [44,45]; three had no age restrictions [27,73], including our current study. All studies included both male and female participants. Four studies [27,73,74], including ours, categorised participants according to their average Phe levels, while three studies [44,45], including ours, used the level of formula consumption as the basis for grouping. One study [45] included both classical and mild forms of PKU, whereas two others [28,44] did not clarify the PKU subtype among their participants. From one study [73], we extracted only data from patients diagnosed with classical PKU. The remaining studies focused on individuals with classical PKU. Table 6 and Table 7 provide an overview of the participants’ dietary habits and metabolic profiles.

#### 3.2.3. Risk of Bias

Table 8 summarises the bias assessment results, showing cross-sectional studies scored between 3 and 9 points, reflecting high to low risk of bias. Regarding selection bias, three studies [27,74] (+present study) (42.9%) were awarded a point for sample representativeness, while only one study justified the sample size (present study). Blood samples were collected from all participants invited, indicating a full response rate across all studies. All studies used validated tools to measure vitamin levels. Regarding comparability, 71.4% of studies controlled for at least one key confounder. Outcome assessment, based on the NOS criteria, awards points only if blinded evaluation, record linkage, or self-reporting was used. Only one study reported blinded evaluation. Although vitamin measurements are objective, the lack of blinding information limited scoring. Statistical analysis was generally adequate across studies, but most did not report confidence intervals, which reduced scores in the statistical domain.

#### 3.2.4. Comparison of Vitamin D Levels in Adherent vs. Non-Adherent Individuals

**Figure 3:**
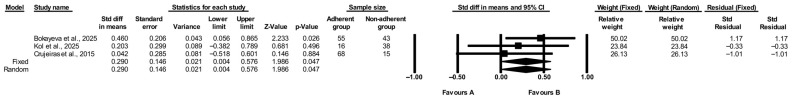
Forest plot of vitamin D levels in adherent individuals (favours B) vs. non-adherent individuals (favours A) (fixed model). CI—confidence interval; Std—standard; Std diff—standard differences. The analysis includes 2 published papers [73,74] and the results of the present study (Bokayeva et al., 2025).

Vitamin D levels were assessed in three studies [73,74] (+present study) (Table 9). Pooled data demonstrated that adherent individuals on the PKU diet had significantly higher vitamin D levels than non-adherent individuals. The meta-analysis yielded an SMD of 0.290 (fixed-effects model: 95% CI: 0.004, 0.576, *p* = 0.047) (Figure 3). Heterogeneity across studies was low (Q = 1.525, *p* = 0.466, *I*^2^ = 0.0%). Cumulative analysis is presented in Figure S1. The 2015 study by Crujeiras et al. [73] showed no significant effect (*p* = 0.884), whereas the 2025 studies by Kol et al. [74] and the present study demonstrated a trend toward significance (*p* = 0.047). Sensitivity analysis (Figure S2) showed that the individual studies strongly influenced the cumulative effect. Removing the Crujeiras et al. [73] study slightly strengthened the association (*p* = 0.026), whereas excluding our present study rendered the result non-significant (*p* = 0.565). Excluding the Kol et al. [74] paper yielded a borderline effect (*p* = 0.058), indicating that the most recent studies primarily drive the significant association.

**Figure 4:**
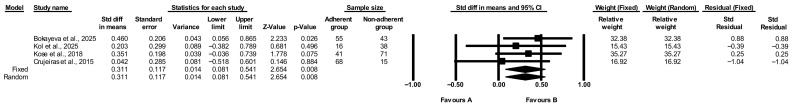
Forest plot of vitamin D levels in adherent individuals (favours B) vs. non-adherent individuals (favours A) (fixed model), expanded analysis. CI—confidence interval; Std—standard; Std diff—standard differences. The analysis includes 3 published papers [27,73,74] and the results of the present study (Bokayeva et al., 2025).

We conducted additional analyses incorporating one additional study [27]. This study population consisted primarily of patients diagnosed through neonatal screening, with a subset identified via clinical findings. It also applied different age cut-offs, defining high adherence as < 360 μmol/L for patients under 6 years, <480 μmol/L for those 6–10 years, and ≤ 600 μmol/L for older patients, according to Vockley et al. [75]. The expanded meta-analysis demonstrated consistent results, with an SMD of 0.311 (fixed-effects model: 95% CI: 0.081, 0.541, *p* = 0.008) (Figure 4), reinforcing the significant vitamin D advantage in adherent patients. Heterogeneity remained low (Q = 1.588, *p* = 0.662; *I*^2^ = 0.0%). In cumulative analysis (Figure S3), effect sizes increased across studies, from 0.042 (*p* = 0.884) in 2015 to 0.311–0.331 (*p* < 0.01) in 2025, indicating a cumulative trend toward a significant association over time. Sensitivity analysis is presented in Figure S4. Removal of the present study (Bokayeva et al.) rendered the result non-significant (*p* = 0.092), whereas excluding the Kol et al. [74] study maintained significance (*p* = 0.009). Excluding Kose et al. [27] study or the Crujeiras et al. The [73] study showed slight reduction in the overall effect but the results remained significant (*p* = 0.047 and *p* = 0.004, respectively). This suggests that the significant cumulative effect is largely driven by the present study, with additional contributions from the other studies. After removing studies with a high risk of bias, vitamin D levels remained significantly higher in adherent versus non-adherent PKU patients (fixed-effects model SMD: 0.404, 95% CI: 0.124, 0.683; *p* = 0.005) (Figure S5).

#### 3.2.5. Comparison of Vitamin D Levels in Regular Intake vs. Irregular Formula Intake Individuals

**Figure 5:**
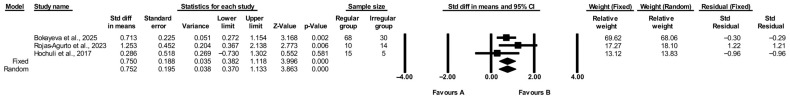
Forest plot of vitamin D levels in regular formula intake individuals (favours B) vs. irregular intake individuals (favours A) (fixed model). CI—confidence interval; Std—standard; Std diff—standard differences. The analysis includes 2 published papers [44,45] and the results of the present study (Bokayeva et al., 2025).

Three studies [44,45] (+present study) were included in the meta-analysis comparing vitamin D levels between individuals with regular versus irregular formula intake (Table 10). Our assessment revealed that individuals with PKU who maintained regular formula intake had significantly higher vitamin D levels compared to those with irregular intake (fixed-effects model: SMD = 0.750, 95% CI: 0.382, 1.118, *p* < 0.0001) (Figure 5). Between-study heterogeneity was low (Q = 2.066, *p* = 0.356, *I*^2^ = 3.210%). Effect sizes in cumulative analysis (Figure S6) increased across the three studies, from 0.286 (*p* = 0.581) in 2017 to 0.836 (*p* = 0.014) in 2023 and 0.750 (*p* < 0.0001) in 2025, demonstrating a cumulative trend toward a significant and robust association in recent studies. Sensitivity analysis (Figure S7) showed that the association remained statistically significant regardless of which study was removed.

After removing the study with a high risk of bias [45], the difference in vitamin D levels between groups remained statistically significant (SMD = 0.820, 95% CI: 0.425, 1.215, *p* < 0.0001) (Figure S8). The heterogeneity was low (Q = 1.145, *p* = 0.285; *I*^2^ = 12. 631%).

#### 3.2.6. Comparison of Vitamin E Levels in Adherent vs. Non-Adherent Individuals

**Figure 6:**
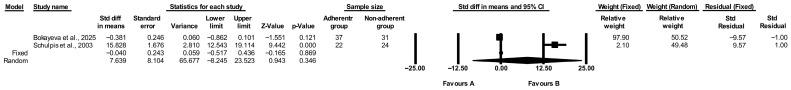
Forest plot of vitamin E levels in adherent individuals (favours B) vs. non-adherent individuals (favours A) (random-effects model). CI—confidence interval; Std—standard; Std diff—standard differences. The analysis includes 1 published paper [28] and the results of the present study (Bokayeva et al., 2025).

The meta-analysis of two included studies [28] (+present study) revealed no statistically significant difference in vitamin E levels between adherent and non-adherent PKU individuals (random-effects model: SMD = 7.639, 95% CI: −8.245, 23.523, *p* = 0.346) (Figure 6). Heterogeneity analysis indicated high variation across the included studies (Q = 91.525, *p* = 0, *I*^2^ = 98.907%). The results of cumulative analysis are presented in Figure S9. While the 2003 study [28] reported a highly significant effect, the present study (Bokayeva et al.) showed a non-significant effect. This discrepancy confirms high heterogeneity across studies. Sensitivity analysis (Figure S10) revealed that the significant cumulative effect was almost entirely attributable to Schulpis et al. [28]’s study. Excluding the present research (Bokayeva et al.) yielded a highly significant result (*p* < 0.0001), whereas excluding of Schulpis et al. [28] rendered the association non-significant (*p* = 0.121).

#### 3.2.7. Comparison of Beta-Carotene Levels in Adherent vs. Non-Adherent Individuals

**Figure 7:**
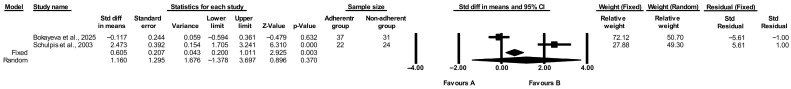
Forest plot of beta-carotene levels in adherent individuals (favours B) vs. non-adherent individuals (favours A) (random-effects model). CI—confidence interval; Std—standard; Std diff—standard differences. The analysis includes 1 published paper [28] and the results of the present study (Bokayeva et al., 2025).

Pooled data from two studies [28] (+present study) demonstrated no statistically significant difference in beta-carotene levels between adherent and non-adherent PKU patients (random-effects model: SMD = 1.160, 95% CI: −1.378, 3.697, *p* = 0.370) (Figure 7), with high heterogeneity (Q = 31.487, *p* < 0.0001, *I*^2^ = 96.824%). The cumulative analysis results are shown in Figure S11, while the sensitivity analysis findings are depicted in Figure S12. Schulpis et al. [28] reported a substantial and statistically significant effect, whereas the present study (Bokayeva et al.) showed a smaller, non-significant effect, confirming heterogeneity across studies.

#### 3.2.8. Certainty of Evidence Assessment

Table 11 and Table 12 summarise the GRADE certainty assessments for outcomes comparing vitamin A, D, E and beta-carotene across the investigated groups. The certainty of evidence across outcomes evaluating vitamin status in individuals with PKU varied from very low to low, primarily due to limitations in study quality, inconsistency, and imprecision.

For vitamin D, both comparisons (adherent vs. non-adherent; regular vs. irregular intake) showed statistically significant effects, with low heterogeneity and consistent direction and magnitude of effects across studies. In the expanded analysis, including four studies, the evidence was downgraded for risk of bias, primarily due to moderate-quality studies and sensitivity results indicating borderline statistical significance when excluding one study. In the analysis comparing regular vs. irregular intake, the evidence was downgraded for imprecision due to small sample sizes, despite the significant and precise pooled estimate. For vitamin E and beta-carotene, the certainty of evidence was rated very low due to both severe inconsistency (*I*^2^ exceeding 95%) and very serious imprecision, with wide confidence intervals. The meta-analyses for these outcomes included only two studies each, further limiting the ability to explore heterogeneity or draw firm conclusions. Regarding risk of bias, several studies lacked information on control for confounding factors, and in one case, removing a single study from the analysis altered the statistical significance of the result. Begg’s and Egger’s tests for publication bias were performed; however, given that most meta-analyses included fewer than 10 studies, these statistical tests are not reliable for detecting publication bias. Therefore, no downgrades were applied on this basis.

## 4. Discussion

We combined cross-sectional data with a systematic review and meta-analysis to explore how adherence to dietary treatment and the regular use of Phe-free formula affect fat-soluble vitamin levels in people with PKU. Overall, the results indicate that vitamin D status is influenced by treatment adherence and formula use. In contrast, vitamins A and E are well maintained and beta-carotene shows only modest, formula-related differences.

The most precise and most consistent finding across our analyses concerns vitamin D. Both the cross-sectional data and pooled meta-analytic results showed that individuals adhering to dietary recommendations had significantly higher serum 25(OH) D levels compared to non-adherent individuals (fixed-effects model: SMD = 0.290, 95% CI: 0.004, 0.576, *p* = 0.047) This association persisted even after removing high-risk-of-bias studies in extended analysis. Notably, the effect size was even larger in patients with regular formula intake (SMD = 0.750, 95% CI: 0.382, 1.118, *p* < 0.0001), underscoring the critical role of formulas in meeting nutritional needs.

Ensuring adequate vitamin D intake is challenging even for healthy individuals [76], but the restrictive diet in PKU makes it particularly problematic. The usual food sources (fatty fish, egg yolks, beef liver, dairy products) are either restricted or entirely excluded due to their high Phe content [6,30]. Although sunlight exposure can support endogenous vitamin D synthesis, various factors, including geographic location, skin pigmentation, time of day, season of the year, latitude, ageing, sunscreen use and modern indoor lifestyles often limit its effectiveness [77]. Overweight and obesity are commonly reported in children and adults with classical PKU, particularly among females in some cohorts [78,79,80,81]. In this context, increased body fat may further lower circulating 25(OH)D concentrations [82,83,84] and thus increase the overall risk of vitamin D deficiency in this population. The combination of a low vitamin D diet, reliance on protein substitutes, and the tendency toward increased adiposity in the PKU population means these individuals are particularly vulnerable to suboptimal vitamin D status. Consequently, PKU patients rely primarily on nutrient-enriched formulas, which appear to be the most consistent contributor to maintaining adequate vitamin D levels.

In our cross-sectional analysis, the majority of adherent individuals (63.6% vs. 51.2%) and regular formula users (60.3% vs. 53.3%) had optimal vitamin D levels, whereas suboptimal levels were more frequent in non-adherent individuals (21.8% vs. 40.5%) and those with irregular intake (25% vs. 43.3%). High concentrations were more frequent among adherent individuals (19.2%) and regular formula users (18.8%) than among non-adherent individuals (4.9%) and irregular users, for whom no high levels were observed. The only participant with vitamin D deficiency was a non-adherent and irregular formula consumer. The frequency of vitamin D deficiency and suboptimal levels is more common in non-adherent patients (44.2% vs. 21.8% in adherent; *p* = 0.018) and in those with irregular formula intake (46.7% vs. 25.0% in regular users; *p* = 0.034). Although the deficiency was rare in this cohort, “low” levels were substantially more frequent among non-adherent individuals and irregular formula users, highlighting the importance of dietary management for maintaining sufficient vitamin D status.

Our findings are consistent with those of Rojas-Agurto et al. [44], who reported significantly lower vitamin D levels in individuals with PKU who had discontinued protein substitutes compared with those who remained on dietary treatment. In contrast, Kose et al. [27] did not observe a statistically significant difference in mean vitamin D levels between adherent and non-adherent participants. However, they did report a negative correlation (r = −0.309, *p* = 0.001) between plasma Phe levels and 25(OH)D, suggesting an indirect relationship between metabolic control and vitamin D status. A recent study in early and continuously treated adults with PKU reported that vitamin D concentrations were significantly higher in patients than in matched controls, and that protein substitutes provided almost 90% of patients’ vitamin D intake and more than half of their total micronutrient intake, thereby essentially supporting adequate vitamin D status despite variable dietary adherence [85]. A retrospective study of children with inborn errors of metabolism, including PKU, consuming medical food-based diets, reported normal 25(OH)D concentrations and bone mineral density. Authors found out that the overall risk of vitamin D deficiency is similar to that of the general population, suggesting that appropriately fortified medical foods can effectively secure vitamin D status when taken as prescribed [86]. Our previous meta-analysis [42] revealed that PKU patients tend to have higher levels of 1,25-dihydroxyvitamin D than controls, though 25(OH)D levels were not significantly different. Leiva et al. [41] found that PKU patients who continued treatment with protein substitutes had higher vitamin D levels and comparable spine and femoral neck bone mineral density to healthy controls, highlighting the role of adequate vitamin D intake in supporting bone health. When protein substitutes are used as prescribed, individuals with PKU can achieve vitamin D levels comparable to or even better than those of the general population.

Vitamin A levels were elevated in a small proportion of individuals across all groups, with no cases of deficiency observed. In the highest quartile of serum vitamin A (Q4), concentrations ranged from 724 to 960 ng/mL in adherent participants and from 740 to 947 ng/mL in non-adherent participants; elevated values occurred in 5 of 38 adherent (13.2%) and 5 of 31 non-adherent individuals (16.0%). Similarly, Q4 values ranged from 724.5 to 960.0 ng/mL in regular formula users and from 718 to 947 ng/mL in irregular users, with elevated vitamin A observed in 8 of 44 regular (18.2%) and 2 of 25 irregular consumers (8.0%). All values remained below 1000 ng/mL, a concentration often used as a biochemical threshold for hypervitaminosis A [87,88], and no clinical signs of toxicity were observed. This suggests no meaningful variation in vitamin A status by adherence or formula intake. No significant differences in vitamin A were observed between groups in our cross-sectional study. The meta-analysis for vitamin A was not feasible.

All participants had vitamin E levels within the reference range, regardless of adherence or formula intake. The concentrations did not differ significantly between groups in the cross-sectional part of the study. The meta-analysis of vitamin E did not show a statistically significant difference between adherent and non-adherent individuals (random-effects model: SMD = 7.639, 95% CI: −8.245, 23.523, *p* = 0.346). Since the analysis was based on only two studies with substantial heterogeneity and wide confidence intervals, the pooled vitamin E results should be interpreted with particular caution.

In our cross-sectional analysis, beta-carotene status was similar across adherence groups, but its levels were significantly higher in individuals with regular formula intake (74.40 [56.70–98.45] vs. 53.20 [34.10–68.60] ng/mL, *p* = 0.003). Most participants across groups had concentrations within the reference range, and deficiency was relatively uncommon and did not differ between groups. It was observed in 20.0% of individuals with irregular formula intake, compared with 7.0% in those with regular intake. The meta-analysis for beta-carotene showed no statistically significant difference between adherent and non-adherent individuals (random-effects model: SMD = 1.160, 95% CI: −1.378, 3.697, *p* = 0.370). Given that this estimate is based on two highly heterogeneous studies and is accompanied by wide confidence intervals, the pooled beta-carotene results should be interpreted as exploratory rather than conclusive.

The present findings suggest that fat-soluble vitamin status in PKU is relatively well maintained. The lack of differences in vitamins A and E between groups may reflect the body’s capacity to store these nutrients [7,89,90] and their presence in both fortified products and some low-Phe products permitted in the PKU diet. Since beta-carotene from plant sources is converted to active vitamin A via the intestinal BCMO1 enzyme [7,9], this pathway could partially explain the stable vitamin A levels across groups. Similarly, vitamin E—derived from vegetable oils, butter, tomato, leafy greens [6,19,20]—may be sufficiently supplied through both allowed foods and formula, leading to uniformly adequate levels regardless of adherence. Interestingly, beta-carotene levels were significantly higher among regular formula users in our cross-sectional study, despite beta-carotene not being commonly added to Phe-free formula. Regular intake of Phe-free formula provides sufficient vitamin A, which may reduce the conversion of dietary beta-carotene to active vitamin A [91] and thereby contribute to higher circulating beta-carotene levels in regular formula users. Regular formula use may improve overall fat intake. Oils and fats, which are commonly added to formula, can improve carotenoid bioaccessibility by helping to disperse carotenoids, which facilitates their solubilisation and emulsification during digestion [92]. Schulpis et al. [28] reported that PKU patients adhering to a strict diet had significantly higher blood levels of vitamin E and beta-carotene, along with increased total antioxidant status compared to non-adherent individuals. They also found positive correlations between antioxidant vitamin levels and plasma antioxidant capacity, particularly in the adherent group. Similarly, Sanayama et al. [16] reported a significant negative correlation between beta-carotene and serum Phe levels (r = −0.421, *p* < 0.05), further supporting the association between better metabolic control and higher antioxidant nutrient levels. This is particularly relevant, as Phe has been shown in vitro to reduce synaptic density and impair synaptic activity in rat hippocampal neurons [93], potentially via oxidative stress [94,95]. Maintaining adequate intake of antioxidants, such as beta-carotene and vitamin E, may help mitigate oxidative effects in individuals with PKU.

In summary, vitamin D emerges as the fat-soluble vitamin most clearly linked to adherence to the Phe-free formula. In contrast, vitamins A and E are generally maintained within reference ranges across all adherence categories. This stronger association for vitamin D likely reflects its heavy reliance on fortified medical formulas and supplements within the PKU diet. Consequently, in current dietary management, regular consumption of protein substitutes is critical for ensuring adequate vitamin D status. In contrast, vitamins A and E appear less dependent on formula adherence, as their substantial body stores and presence in both fortified products and permitted natural foods may buffer variations in intake.

Our previous systematic review and meta-analysis [42] showed that individuals with PKU typically have vitamin A, vitamin E and 25(OH)D concentrations comparable to those of controls, while 1,25-dihydroxyvitamin D levels are higher (high heterogeneity; no significant difference after excluding a high risk-of-bias study). Together with the current findings, this suggests that overall fat-soluble vitamin status in PKU is broadly similar to that of the general population. Still, within the PKU population, vitamin D remains particularly sensitive to adherence and regular formula use.

From a clinical perspective, these results support maintaining protein substitutes as a central component of PKU management and prioritising systematic monitoring of fat-soluble vitamins, especially vitamin D and A. In nutritional management, particular emphasis should be placed on the possibility of excessive vitamin A intake.

To the best of our knowledge, the study represents the first meta-analysis to comprehensively evaluate differences in vitamins A, D, E and beta-carotene status between: 1. PKU patients with strict dietary adherence versus those with poor adherence, and 2. individuals maintaining regular versus irregular intake of metabolic formulas. By integrating original data from cross-sectional research with a comprehensive systematic review and meta-analysis, we provide a more robust evidence base than either approach could offer on its own. Our findings are supported by an appropriately powered study population and well-defined criteria for assessing both dietary adherence and formula intake consistency. We carefully implemented eligibility criteria, specifically selecting participants with classic PKU diagnosed through neonatal screening who were receiving consistent treatment, thereby reducing potential confounding variables. A significant strength of the meta-analysis lies in its rigorous methodology, including strict inclusion/exclusion criteria, group division, and sensitivity analyses. We adhered to PRISMA guidelines and Cochrane Handbook recommendations and applied statistical methods to account for heterogeneity and risk of bias. However, several limitations must be acknowledged. First, the number of eligible studies was limited, which prevented us from performing subgroup analyses. Second, high heterogeneity was observed in some comparisons (e.g., *I*^2^ > 90% for vitamin E and beta-carotene). Third, including both paediatric and adult participants in our study population may introduce some variability in results due to potential age-related metabolic differences. Moreover, the lack of standardised criteria for categorising formula consistency across studies introduces potential measurement bias that may affect outcome comparisons. Although mean Phe levels reflect overall dietary control, they provide incomplete information about formula intake patterns, which are essential for nutrient sufficiency. The limited number of studies available for specific analyses may reduce statistical power and affect the generalisability of these findings. Additionally, in the cross-sectional study, some participants reported vitamin D supplementation. Overall, 14 out of 98 participants (14%) were taking vitamin D supplements. Given the relatively low prevalence of supplementation, it is unlikely to have been entirely responsible for the observed vitamin D status, but it is essential to note this. Considering limitations, the obtained results should be interpreted with appropriate caution.

Our bias assessment revealed several limitations that warrant consideration when interpreting the findings. Selection bias concerns arose from issues of representativeness and sample size. In the outcome assessment domain, only one study reported blinding procedures, though this may be less critical given the objective nature of vitamin level measurements. In the statistical domain, most studies lacked confidence intervals, affecting their scores despite otherwise adequate analyses. The NOS itself has limitations, including reliance on subjective judgement and a focus on study design. Additionally, the NOS lacks quantitative bias measurement. Finally, publication bias could not be reliably assessed due to the small number of included studies.

Our findings may have been influenced by small sample sizes, which may have limited statistical power. Inconsistent definitions of formula intake across studies may have introduced misclassification bias. Our classification method may have introduced variability in the observed vitamin levels, as plasma Phe concentrations, while indicative of dietary adherence, can also be affected by individual metabolic differences and factors such as PKU subtype. Unmeasured confounding variables, such as sun exposure or genetic differences in nutrient metabolism, may have affected individual vitamin statuses but were not reported across studies. Potential changes in formula composition over the years may have affected the results. Variations in patient demographics (age, sex, and ethnicity) across the included studies may have also contributed to heterogeneity in the results. Including studies with unspecified or mixed PKU types may have contributed to variability, as not all studies specifically focused on individuals with classical PKU.

Meta-regression, network meta-analysis, and subgroup analyses based on factors such as sex, type of protein substitute, or metabolic control were not feasible due to the limited number of eligible studies and missing data. Given the very low to low quality of evidence, as assessed using the GRADE approach, the findings of this meta-analysis should be interpreted with caution. Larger, well-designed studies are necessary to confirm these results.

## 5. Conclusions

Adherence to diet and regular formula intake is associated with improved vitamin D status, underscoring the critical role of fortified formulas in PKU management. The very low certainty of evidence necessitates further research, especially for the other fat-soluble vitamins. Nonetheless, clinical practice should emphasise support for adherence and ongoing nutritional monitoring.

## Figures and Tables

**Table 1:** Interpretation of 25-hydroxyvitamin D (25(OH)D) levels.

25(OH)D Level (ng/mL)	Interpretation
0–10	Severe deficiency
>10–20	Significant deficiency
20–30	Insufficient/Suboptimal
30–50	Optimal
>50–75	High but not toxic
>75–100	Very high
>100	Toxic

**Table 2:** Demographic, anthropometric, metabolic and biochemical parameters of the adherent and non-adherent PKU groups.

Parameter	Adherent PKU Group Median (Q1–Q3); Mean ± SD	Non-Adherent PKU Group Median (Q1–Q3); Mean ± SD	*p*-Value
**Smaller cohort (*n* = 68)**			
*n*	37	31	
Age (years)	19 (14.00–27.80); 20.65 ± 7.31	19.9 (16.20–28.75); 22.78 ± 8.82	0.345 ^1^
Sex (*n* (%)) Female Male	23 (62.2%) 14 (37.8%)	15 (48.4%) 16 (51.6%)	0.254 ^2^
BMI (kg/m^2^)	21.90 (19.80–24.09); 21.59 ± 3.42	21.68 (19.77–28.77); 24.16 ± 5.51	0.161 ^1^
BMI-IOTF corrected (kg/m^2^)	23.44 (19.84–24.76); 22.61 ± 3.64	22.91 (21.07–28.77); 24.85 ± 5.07	0.144 ^1^
Phe mean (mg/dL)	7.21 (5.50–8.85); 6.88 ± 2.27	14.56 (12.23–17.41); 15.03 ± 3.84	<0.0001 ^4^*
Phe median (mg/dL)	6.50 (5.09–8.54); 6.50 ± 2.44	14.56 (11.93–17.54); 14.90 ± 3.76	0.0001 ^1^*
Abnormal Phe values (%)	23.7 (4.4–33.3); 20.5 ± 15.1	100 (78.6–100); 88.8 ± 14.6	0.0001 ^1^*
Vitamin A (ng/mL)	655 (549–724); 641.2 ± 146.9 95% CI (592.2–690.2)	637 (571–740); 652.2 ± 145.6 95% CI (598.8–705.6)	0.758 ^3^
Vitamin E (µg/mL)	9.90 (8.80–11.30); 10.16 ± 1.62 95% CI (9.63–10.70)	10.50 (9.30–12.30); 10.90 ± 2.27 95% CI (10.07–11.73)	0.125 ^3^
Beta-carotene (ng/mL)	61.90 (48.80–87.90); 74.00 ± 53.73 95% CI (56.09–91.92)	62.80 (43.55–87.80); 80.58 ± 59.43 95% CI (58.78–102.38)	0.696 ^1^
**Larger cohort (*n* = 98)**			
*n*	55	43	
Age (years)	16.6 (12.35–27.40); 19.40 ± 8.73	19.1 (15.45–26.70); 21.39 ± 8.74	0.206 ^1^
Sex (*n* (%)) Female Male	30 (54.5%) 25 (45.5%)	21 (48.8%) 22 (51.2%)	0.575 ^2^
BMI (kg/m^2^)	21.52 (18.18–24.10); 21.34 ± 4.22	21.81 (19.76–27.47); 23.59 ± 5.11	0.075 ^1^
BMI-IOTF corrected (kg/m^2^)	23.44 (20.54–24.81); 23.11 ± 4.46	22.91 (21.07–27.84); 24.60 ± 4.87	0.219 ^1^
Phe mean (mg/dL)	6.30 (4.85–8.85); 6.58 ± 2.37	13.41 (11.38–17.07); 14.17 ± 3.87	0.0001 ^1^*
Phe median (mg/dL)	6.33 (3.93–8.49); 6.21 ± 2.54	13.18 (11.11–17); 14.05 ± 3.77	0.0001 ^1^*
Abnormal values (%)	21.4 (6.8–33.7); 21.5 ± 15.1	88.9 (73.3–100); 83.8 ± 19.3	0.0001 ^1^*
Vitamin D (ng/mL)	35.60 (30.39–41.65); 37.92 ± 10.33 95% CI (35.13–40.71)	32.90 (26.50–40.00); 33.48 ± 8.68 95% CI (30.81–36.15)	0.034 ^1^*

*—statistically significant; ^1^—Mann–Whitney U-test; ^2^—Pearson’s Chi-square test, ^3^—Student’s *t*-test; ^4^—Welch’s *t*-test; significance level—0.05; BMI—body mass index, CI—confidence interval, IOTF—International Obesity Task Force, SD—standard deviation.

**Table 3:** Demographic, anthropometric, metabolic and biochemical parameters of PKU patients with regular and irregular intakes of formula.

Parameter	Regular PKU Group Median (Q1–Q3); Mean ± SD	Irregular PKU Group Median (Q1–Q3); Mean ± SD	*p*-Value
**Smaller cohort (*n* = 68)**			
*n*	43	25	
Age (years)	18.4 (13.75–25.55); 19.96 ± 7.37	24.2 (17.0–29.6); 24.48 ± 8.49	0.026 ^1^*
Sex (*n* (%)) Female Male	26 (60.5%) 17 (39.5%)	12 (48%) 13 (52%)	0.318 ^2^
BMI (kg/m^2^) BMI-IOTF corrected (kg/m^2^)	21.36 (19.38–23.44); 21.46 ± 3.64 23.36 (20.06–24.77); 22.59 ± 3.62	24.35 (21.0–29.65); 25.01 ± 5.35 24.35 (21.13–30.19); 25.42 ± 5.24	0.005 ^3^* 0.022 ^3^*
Phe mean (mg/dL)	7.96 (5.90–9.89); 8.02 ± 3.21	16.66 (11.65–18.70); 15.02 ± 4.76	<0.0001 ^3^*
Phe median (mg/dL)	7.94 (5.59–9.86); 7.79 ± 3.33	15.91 (11.36–17.73); 14.70 ± 5.04	<0.0001 ^3^*
Abnormal values (%)	33.3 (7.6–50.9); 35.7 ± 32.3	100 (66.8–100); 79.0 ± 28.7	0.0001 ^1^*
Vitamin A (ng/mL)	663 (564.5–724.5); 661.0 ± 140.9 95% CI (617.6–704.4)	623 (516–718); 620.8 ± 152.1 95% CI (558–683.6)	0.274 ^4^
Vitamin E (µg/mL)	9.90 (9.20–11.55); 10.39 ± 1.64 95% CI (9.89–10.90)	10.20 (8.9–12.1); 10.68 ± 2.45 95% CI (9.67–11.69)	0.559 ^4^
Beta-carotene (ng/mL)	74.40 (56.70–98.45); 89.67 ± 63.29 95% CI (70.19–109.15)	53.20 (34.10–68.60); 55.21 ± 31.35 95% CI (42.27–68.15)	0.003 ^1^*
**Larger cohort (*n* = 98)**			
*n*	68	30	
Age (years)	16.65 (12.10–24.53); 18.88 ± 8.44	22.8 (15.75–29.38); 23.43 ± 8.74	0.015 ^1^*
Sex (*n* (%)) Female Male	36 (52.9%) 32 (47.1%)	15 (50%) 15 (50%)	0.788 ^2^
BMI (kg/m^2^) BMI-IOTF corrected (kg/m^2^)	21.16 (17.96–23.44); 21.15 ± 3.80 22.84 (20.54–24.79); 22.96 ± 4.11	24.16 (21.10–29.03); 25.00 ± 5.60 24.61 (21.20–30.06); 25.58 ± 5.41	0.002 ^1^* 0.031 ^1^*
Phe mean (mg/dL)	7.93 (4.96–10.11); 7.99 ± 3.56	13.77 (10.03–17.51); 14.25 ± 4.76	<0.0001 ^3^
Phe median (mg/dL)	7.86 (5.08–9.97); 7.72 ± 3.67	14.34 (10.41–17.56); 14.03 ± 4.92	<0.0001 ^3^
Abnormal values (%)	33.3 (12.0–50.4); 36.8 ± 31.3	85.2 (62.1–100); 76.3 ± 28.3	0.0001 ^1^*
Vitamin D (ng/mL)	35.97 (30.03–42.28); 38.02 ± 10.38 95% CI (35.51–40.54)	30.20 (26.08–35.06); 31.32 ± 6.59 95% CI (28.86–33.78)	0.002 ^1^*

*—statistically significant; ^1^—Mann–Whitney U-test; ^2^—Pearson’s Chi-square test; ^3^—Welch’s *t*-test; ^4^—Student’s *t*-test; significance level—0.05; BMI—body mass index, CI—confidence interval, IOTF—International Obesity Task Force, SD—standard deviation.

**Table 4:** Characteristics of included studies and studied individuals: adherent vs. non-adherent.

Author	Year	Country (Region)	Groups	*n* Included	*n* Completed	Age [Years] ^1^	BMI [kg/m^2^] ^1^	Sex [% of Women]
Bokayeva et al.	2025	Poland	Adherent ^2^	37	37	20.65 ± 7.31	21.59 ± 3.42	62.2
			Non-adherent ^2^	31	31	22.78 ± 8.82	24.16 ± 5.51	48.4
			Adherent ^3^	55	55	19.40 ± 8.73	21.34 ± 4.22	54.5
			Non-adherent ^3^	43	43	21.39 ± 8.74	23.59 ± 5.11	48.8
Kol et al. [74]	2025	Turkey	Adherent	16	16	10.7 ± 3.7	NI	44.4
			Non-adherent	38	38			
Kose et al. [27]	2018	Turkey	Adherent	41	41	11.4 ± 6.8	NI	47.3
			Non-adherent	71	71			
Crujeiras et al. [73]	2015	Spain	Adherent	68	68	10.97 (1–92) ^4^	NI	47.8
			Non-adherent	15	15	22.27 (3–30) ^4^		50
Schulpis et al. [28]	2003	Greece	Adherent	22	22	7.7 ± 3.2	NI	NI
			Non-adherent	24	24	8.0 ± 3.6		

^1^—mean ± standard deviation; ^2^—the group with vitamin A, E, and beta-carotene measurements (cohort S); ^3^—the group with vitamin D measurements (cohort L); ^4^—mean (min–max). BMI—body mass index; NI—no information.

**Table 5:** Characteristics of included studies and studied individuals: regular vs. irregular.

Author	Year	Country (Region)	Groups	*n* Included	*n* Completed	Age [Years] ^1^	BMI [kg/m^2^] ^1^	Sex [% of Women]
Bokayeva et al.	2025	Poland	Regular ^2^	43	43	19.96 ± 7.37	21.46 ± 3.64	60.5
			Irregular ^2^	25	25	24.48 ± 8.49	25.01 ± 5.35	48
			Regular ^3^	68	68	18.88 ± 8.44	21.15 ± 3.80	52.9
			Irregular ^3^	30	30	23.43 ± 8.74	25.00 ± 5.60	50
Rojas-Agurto et al. [44]	2023	Chile	Regular ^4^	10	10	23.5 (19–26) ^6^	24.3 (22.4–28.5) ^6^	50
			Irregular ^5^	14	14	22.5 (18.5–25.5) ^6^	26.7 (24–29.9) ^6^	36
Hochuli et al. [45]	2017	Switzerland	Regular ^7^	15	15	32 ± 12	24.6 ± 4.3	53
			Irregular ^8^	5	5	39 ± 8.4	20.6 ± 2.1	20

^1^—mean ± standard deviation; ^2^—the group with vitamin A, E, and beta-carotene measurements (cohort S); ^3^—the group with vitamin D measurements (cohort L); ^4^—patients under diet treatment; ^5^—patients who discontinued the protein substitution at 18 years of age; ^6^—median (25th–75th centile); ^7^—regular Phe-free amino acid mixture intake; ^8^—Phe-free amino acid mixture intake below the prescribed amount; BMI—body mass index; NI—no information.

**Table 6:** Characteristics of diet and metabolic status of studied individuals: adherent vs. non-adherent.

Author	Year	Groups	Phe Intake [mg/d]	Mean Phe Levels	Medical Control	Last Phe [μmol/L] ^1^	Last Tyr [μmol/L] ^1^
Bokayeva et al.	2025	Adherent ^2^	NI	6.88 ± 2.27 ^4^	Yes	NI	NI
		Non-adherent ^2^		15.03 ± 3.84 ^4^	Yes		
		Adherent ^3^		6.58 ± 2.37 ^4^	Yes		
		Non-adherent ^3^		14.17 ± 3.87 ^4^	Yes		
Kol et al. [74]	2025	Adherent	NI	NI	Yes	299.0 ± 77.2	69.1 ± 60.3
		Non-adherent			Yes	813.7 ± 356.6	68.7 ± 44.0
Kose et al. [27]	2018	Adherent	NI	NI	NI	NI	NI
		Non-adherent					
Crujeiras et al. [73]	2015	Adherent	NI	275.1 ± 133.8 ^5^	NI	NI	NI
		Non-adherent		834.9 ± 291.7 ^5^			
Schulpis et al. [28]	2003	Adherent	NI	292 ± 60 ^5^	Yes No	NI	115.3 ± 26.5 45.8 ± 27.5
		Non-adherent		895 ± 54 ^5^			

^1^—mean ± standard deviation; ^2^—the group with vitamin A, E, and beta-carotene measurements (cohort S); ^3^—the group with vitamin D measurements (cohort L); ^4^—mg/dL; ^5^—μmol/L. Phe—phenylalanine; Tyr—tyrosine; NI—no information.

**Table 7:** Characteristics of diet and metabolic status of studied individuals: regular vs. irregular.

Author	Year	Groups	Phe Intake [mg/d]	Mean Phe Levels	Medical Control	Last Phe [μmol/L] ^1^	Last Tyr [μmol/L] ^1^
Bokayeva et al.	2025	Regular ^2^		8.02 ± 3.21 ^4^	Yes		
		Irregular ^2^		15.02 ± 4.76 ^4^	Yes		
		Regular ^3^		7.99 ± 3.56 ^4^	Yes		
		Irregular ^3^		14.25 ± 4.76 ^4^	Yes		
Rojas-Agurto et al. [44]	2023	Regular ^5^	600 (400–800) ^5^	NI	Yes	260.3 (170–642) ^7^	46.6 (33.1–49.7) ^7^
		Irregular ^6^	1200 (500–1700) ^5^		No	781 (636–1035.1) ^7^	35.9 (33.1–55.2) ^7^
Hochuli et al. [45]	2017	Regular ^8^	NI	NI	NI	650 ± 283	NI
		Irregular ^9^				760 ± 350	

^1^—mean ± standard deviation; ^2^—the group with vitamin A, E, and beta-carotene measurements (cohort S); ^3^—the group with vitamin D measurements (cohort L); ^4^—mg/dL; ^5^—patients under diet treatment; ^6^—patients who discontinued the protein substitution at 18 years of age; ^7^—median (25th–75th centile); ^8^—regular Phe-free amino acid mixture intake; ^9^—Phe-free amino acid mixture intake below the prescribed amount; Phe—phenylalanine; Tyr—tyrosine; NI—no information.

**Table 8:** Newcastle-Ottawa quality assessment scale.

Study (First Author)	Selection				Comparability	Outcome		Overall Score
	Representativeness of the Sample	Sample Size	Non-Respondents	Ascertainment of Exposure	Based on Design and Analysis	Assessment of Outcome	Statistical Test	
Bokayeva et al., 2025 ^1,2^	*+*	+	+	++	+	++	+	9
Kol et al., 2025 [74] ^1^	*+*		+	++				4
Rojas-Agurto et al. [44], 2023 ^2^			+	++	++			5
Kose et al. [27], 2018 ^1^	*+*		+	++	+			5
Hochuli et al. [45], 2017 ^2^			+	++	+			4
Crujeiras et al. [73], 2015 ^1^			+	++				3
Schulpis et al. [28], 2003 ^1^			+	++	+ +			5

^1^—adherence-based groups; ^2^—formula intake regularity groups; +—1 point awarded, ++—2 points awarded.

**Table 9:** Comparison of vitamin status in studied individuals: adherent vs. non-adherent.

Author	Year	Vitamin A ^1^	Beta-Carotene ^1^	Vitamin E ^1^	Vitamin D ^1^
Bokayeva et al.	2025	641.2 ± 146.9 ^2,3^	74.00 ± 53.73 ^2,3^	10.16 ± 1.62 ^2,4^	37.92 ± 10.33 ^2,3,5^
		652.2 ± 145.6 ^2,3^	80.58 ± 59.43 ^2,3^	10.90 ± 2.27 ^2,4^	33.48 ± 8.68 ^2,3,5^
Kol et al. [74]	2025	NA	NA	NA	27.5 ± 9.9 ^2,3,5^
					25.5 ± 9.8 ^2,3,5^
Kose et al. [27]	2018	50.3 (28.9–132.8) ^6,7^	NA	1.3 (0.5–3.3) ^6,8^	20.9 ± 7.1 ^2,3,5^
		57.4 (33.1–111.9) ^6,7^		1.2 (0.6–2.7) ^6,8^	18.2 ± 8.0 ^2,3,5^
Crujeiras et al. [73]	2015	NA	NA	NA	30.25 ± 8.09 ^2,3,5^
					28.77 ± 7 ^2,3,5^
Schulpis et al. [28]	2003	NA	0.70 ± 0.09 ^9,10^	34.0 ± 0.9 ^9,10^	NA
			0.49 ± 0.08 ^9,10^	22.0 ± 0.6 ^9,10^	

^1^—mean ± standard deviation; ^2^—serum; ^3^—ng/mL; ^4^—µg/mL; ^5^—25(OH)D; ^6^—median (min–max); ^7^—μg/dL; ^8^—mg/dL; ^9^—plasma; ^10^—µmol/L; NA—not analysed.

**Table 10:** Comparison of vitamin status in studied individuals: regular vs. irregular.

Author	Year	Vitamin A ^1^	Beta-Carotene ^1^	Vitamin E ^1^	Vitamin D ^1^
Bokayeva et al.	2025	661.0 ± 140.9 ^2,3^	89.67 ± 63.29 ^2,3^	10.39 ± 1.64 ^2,4^	38.02 ± 10.38 ^2,3,5^
		620.8 ± 152.1 ^2,3^	55.21 ± 31.35 ^2,3^	10.68 ± 2.45 ^2,4^	31.32 ± 6.59 ^2,3,5^
Rojas-Agurto et al. [44]	2023	NA	NA	NA	36.97 ± 9.33 ^2,5,6,7^
					24.3 ± 10.62 ^2,5,6,7^
Hochuli et al. [45]	2017	NA	NA	NA	34 ± 10 ^5,8^
					31 ± 12 ^5,8^

^1^—mean ± standard deviation; ^2^—serum; ^3^—ng/mL; ^4^—µg/mL; ^5^—25(OH)D; ^6^—data were received from authors; ^7^—pg/mL; ^8^—μg/L; NA—not analysed.

**Table 11:** Certainty of evidence assessment for vitamins in adherent vs. non-adherent individuals with PKU.

Certainty Assessment							No. of Patients		Effect	Certainty
Outcome and No. of Studies	Study Design	Risk of Bias	Inconsistency	Indirectness	Imprecision	Other Considerations	Adherence	Non-Adherence	Absolute (95% CI)	
Vitamin D–3	non-randomised studies	very serious ^a^	not serious ^b^	not serious	not serious	all plausible residual confounding would reduce the demonstrated effect	139	96	SMD 0.290 SD higher (0.004 higher to 0.576 higher)	⨁◯◯◯ Very low ^a,b^
Vitamin D (expanded)–4	non-randomised studies	Serious ^c^	not serious ^d^	not serious	not serious	all plausible residual confounding would reduce the demonstrated effect	180	167	SMD 0.311 SD higher (0.081 higher to 0.541 higher)	⨁⨁◯◯ Low ^c,d^
Vitamin E–2	non-randomised studies	not serious	very serious ^e^	not serious	very serious ^f^	all plausible residual confounding would reduce the demonstrated effect	59	55	SMD 7.639 SD higher (8.245 lower to 23.523 higher)	⨁◯◯◯ Very low ^e,f^
Beta-carotene–2	non-randomised studies	not serious	very serious ^g^	not serious	very serious ^h^	all plausible residual confounding would reduce the demonstrated effect	59	55	SMD 1.160 SD higher (1.378 lower to 3.697 higher)	⨁◯◯◯ Very low ^g,h^

^a^—downgraded by 2 level due to very serious risk of bias. Two studies did not have info about confounding factors. Sensitivity analysis showed that excluding a single study changed the pooled effect from significant to non-significant; ^b^—heterogeneity is low (Q = 2.467, *p* = 0.291, *I*^2^ = 18.9%). The direction and magnitude of the effect size are similar among studies; ^c^—not downgraded for risk of bias. Although one study had high risk of bias and sensitivity analysis showed the pooled result became borderline non-significant (*p* = 0.053) when it was excluded, the overall findings remained consistent in direction; ^d^—heterogeneity is negligible (Q = 2.581, *p* = 0.461; *I*^2^ = 0.0%). The direction and magnitude of the effect size are similar among studies; ^e^—downgraded by 2 levels for very serious inconsistency. Heterogeneity was extremely high (Q = 90.165, *p* = 0.000, *I*^2^ = 98.891%), and the confidence interval spanned large opposing effect sizes. Only two studies were included, limiting the ability to explore sources of heterogeneity; ^f^—downgraded by 2 levels for very serious imprecision. The confidence interval was extremely wide (95% CI: −8.061, 23.462), and the total sample size was small (2 studies); ^g^—downgraded by 2 levels for very serious inconsistency. Heterogeneity was extremely high (Q = 32.427, *p* < 0.0001, *I*^2^ = 96.916%), and the confidence interval spanned large opposing effect sizes. Only two studies were included, limiting the ability to explore sources of heterogeneity; ^h^—downgraded by 2 levels for very serious imprecision. The confidence interval was wide (95% CI: −1.428, 3.714), the total sample size was small (2 studies). CI—confidence interval; SMD—standardised mean difference.

**Table 12:** Certainty of evidence assessment for vitamins in regular vs. irregular individuals with PKU.

Certainty Assessment							No. of Patients		Effect	Certainty
Outcome and No. of Studies	Study Design	Risk of Bias	Inconsistency	Indirectness	Imprecision	Other Considerations	Adherence	Non-Adherence	Absolute (95% CI)	
Vitamin D–3	non-randomised studies	not serious	not serious ^a^	not serious	serious ^b^	all plausible residual confounding would reduce the demonstrated effect	93	49	SMD 0.75 SD higher (0.382 higher to 1.118 higher)	⨁◯◯◯ Very low ^a,b^

^a^—heterogeneity is negligible (Q = 1.983, *p* = 0.371, *I*^2^ = 0.0%). The direction and magnitude of the effect size are similar among studies. ^b^—downgraded by 2 level for imprecision. Two included studies had small groups with potential for random error. While the effect was statistically significant, the limited overall sample size reduces confidence in the precision. CI—confidence interval; SMD—standardised mean difference.

## Data Availability

The data presented in this study are available on request from the corresponding author due to privacy.

## Supplementary Materials

The following supporting information can be downloaded at: https://www.mdpi.com/article/10.3390/nu17243932/s1, Figure S1. Cumulative meta-analysis of the vitamin D levels in adherent patients (favours B) vs. non-adherent patients (favours A) (fixed model). CI—confidence interval; Std—standard; Std diff—standard differences. The analysis includes 2 published papers [63,64] and the results of the present study (Bokayeva et al., 2025); Figure S2. Sensitivity analysis by the jack-knife approach presenting mean differences with 95% confidence interval in vitamin D levels between adherent patients (favours B) vs. non-adherent patients (favours A) (fixed model). CI—confidence interval; Std diff—standard differences. The analysis includes 2 published papers [63,64] and the results of the present study (Bokayeva et al., 2025); Figure S3. Cumulative meta-analysis of the vitamin D levels in adherent patients (favours B) vs. non-adherent patients (favours A) (fixed model), expanded analysis. CI—confidence interval; Std—standard; Std diff—standard differences. The analysis includes 3 published papers [21,63,64] and the results of the present study (Bokayeva et al., 2025); Figure S4. Sensitivity analysis by the jack-knife approach, presenting mean differences with 95% confidence interval in vitamin D levels between adherent patients (favours B) vs. non-adherent patients (favours A) (fixed model), expanded analysis. CI—confidence interval; Std diff—standard differences. The analysis includes 3 published papers [21,63,64] and the results of the present study (Bokayeva et al., 2025); Figure S5. Sensitivity analysis presenting mean differences with a 95% confidence interval in vitamin D levels between adherent patients (favours B) vs. non-adherent patients (favours A) (fixed model) after exclusion of studies with an overall high risk of bias. CI—confidence interval; Std—standard; Std diff—standard differences. The analysis includes 1 published paper [21] and the results of the present study (Bokayeva et al., 2025); Figure S6. Cumulative meta-analysis of the vitamin D levels in regular intake patients (favours B) vs. irregular intake (favours A) (fixed model). CI—confidence interval; Std—standard; Std diff—standard differences. The analysis includes 2 published papers [38,39] and the results of the present study (Bokayeva et al., 2025); Figure S7. Sensitivity analysis by the jack-knife approach presenting mean differences with 95% confidence interval in vitamin D levels between adherent patients (favours B) vs. non-adherent patients (favours A) (fixed model). CI—confidence interval; Std diff—standard differences. The analysis includes 2 published papers [38,39] and the results of the present study (Bokayeva et al., 2025); Figure S8. Sensitivity analysis presenting mean differences with a 95% confidence interval in vitamin D levels between adherent patients (favours B) vs. non-adherent patients (favours A) (fixed model) after exclusion of studies with an overall high risk of bias. CI—confidence interval; Std—standard; Std diff—standard differences. The analysis includes 1 published paper [38] and the results of the present study (Bokayeva et al., 2025); Figure S9. Cumulative meta-analysis of the vitamin E levels in regular intake patients (favours B) vs. irregular intake (favours A) (random model). CI—confidence interval; Std—standard; Std diff—standard differences. The analysis includes 1 published paper [22] and the results of the present study (Bokayeva et al., 2025); Figure S10. Sensitivity analysis by the jack-knife approach presenting mean differences with 95% confidence interval in vitamin E levels between adherent patients (favours B) vs. non-adherent patients (favours A) (random model). CI—confidence interval; Std diff—standard differences. The analysis includes 1 published paper [22] and the results of the present study (Bokayeva et al., 2025); Figure S11. Cumulative meta-analysis of beta-carotene levels in regular intake patients (favours B) vs. irregular intake (favours A) (random model). CI—confidence interval; Std—standard; Std diff—standard differences. The analysis includes 1 published paper [22] and the results of the present study (Bokayeva et al., 2025); Figure S12. Sensitivity analysis by the jack-knife approach presenting mean differences with 95% confidence interval in beta-carotene levels between adherent patients (favours B) vs. non-adherent patients (favours A) (random model). CI—confidence interval; Std diff—standard differences. The analysis includes 1 published paper [22] and the results of the present study (Bokayeva et al., 2025).

## Abbreviations

The following abbreviations are used in this manuscript:

25(OH)D	25-Hydroxyvitamin D
BCMO1	Beta-Carotene Monooxygenase 1
BH4	Tetrahydrobiopterin
BMI	Body Mass Index
CI	Confidence Interval
GRADE	Grading of Recommendations, Assessment, Development, and Evaluation
IOTF	International Obesity Task Force
NA	Not Analysed
NI	No Information
NOS	Newcastle-Ottawa Scale
PAH	Phenylalanine Hydroxylase
Phe	Phenylalanine
PKU	Phenylketonuria
PRISMA	Preferred Reporting Items for Systematic Reviews and Meta-Analyses
SD	Standard Deviation
SMD	Standardised Mean Difference
Tyr	Tyrosine

## References

[1] van Wegberg A.M.J., MacDonald A., Ahring K., Bélanger-Quintana A., Beblo S., Blau N., Bosch A.M., Burlina A., Campistol J., Coşkun T. (2025). European Guidelines on Diagnosis and Treatment of Phenylketonuria: First Revision. Mol. Genet. Metab..

[2] Singh R.H., Rohr F., Frazier D., Cunningham A., Mofidi S., Ogata B., Splett P.L., Moseley K., Huntington K., Acosta P.B. (2014). Recommendations for the Nutrition Management of Phenylalanine Hydroxylase Deficiency. Genet. Med..

[3] Macdonald A., Davies P., Daly A., Hopkins V., Hall S.K., Asplin D., Hendriksz C., Chakrapani A. (2008). Does Maternal Knowledge and Parent Education Affect Blood Phenylalanine Control in Phenylketonuria?. J. Hum. Nutr. Diet..

[4] Jurecki E.R., Cederbaum S., Kopesky J., Perry K., Rohr F., Sanchez-Valle A., Viau K.S., Sheinin M.Y., Cohen-Pfeffer J.L. (2017). Adherence to Clinic Recommendations among Patients with Phenylketonuria in the United States. Mol. Genet. Metab..

[5] Office of Dietary Supplements Vitamin A and Carotenoids. https://ods.od.nih.gov/factsheets/VitaminA-HealthProfessional/.

[6] Youness R.A., Dawoud A., ElTahtawy O., Farag M.A. (2022). Fat-Soluble Vitamins: Updated Review of Their Role and Orchestration in Human Nutrition throughout Life Cycle with Sex Differences. Nutr. Metab..

[7] Blaner W.S., Marriott B.P., Birt D.F., Stallings V.A., Yates A.A. (2020). Chapter 5—Vitamin A and Provitamin A Carotenoids. Present Knowledge in Nutrition.

[8] Ross A.C., Caballero B., Cousins R.J., Tucker K.L. (2020). Modern Nutrition in Health and Disease.

[9] Kedishvili N.Y. (2013). Enzymology of Retinoic Acid Biosynthesis and Degradation. J. Lipid Res..

[10] Faustino J.F., Ribeiro-Silva A., Dalto R.F., Souza M.M.d., Furtado J.M.F., Rocha G.d.M., Alves M., Rocha E.M. (2016). Vitamin A and the Eye: An Old Tale for Modern Times. Arq. Bras. Oftalmol..

[11] Lanska D.J., Aminoff M.J., Boller F., Swaab D.F. (2009). Chapter 29—Historical Aspects of the Major Neurological Vitamin Deficiency Disorders: Overview and Fat-Soluble Vitamin A. Handbook of Clinical Neurology.

[12] Erkelens M.N., Mebius R.E. (2017). Retinoic Acid and Immune Homeostasis: A Balancing Act. Trends Immunol..

[13] Clagett-Dame M., Knutson D. (2011). Vitamin A in Reproduction and Development. Nutrients.

[14] Larange A., Cheroutre H. (2016). Retinoic Acid and Retinoic Acid Receptors as Pleiotropic Modulators of the Immune System. Annu. Rev. Immunol..

[15] Miazek K., Beton K., Śliwińska A., Brożek-Płuska B. (2022). The Effect of β-Carotene, Tocopherols and Ascorbic Acid as Anti-Oxidant Molecules on Human and Animal In Vitro/In Vivo Studies: A Review of Research Design and Analytical Techniques Used. Biomolecules.

[16] Sanayama Y., Nagasaka H., Takayanagi M., Ohura T., Sakamoto O., Ito T., Ishige-Wada M., Usui H., Yoshino M., Ohtake A. (2011). Experimental Evidence That Phenylalanine Is Strongly Associated to Oxidative Stress in Adolescents and Adults with Phenylketonuria. Mol. Genet. Metab..

[17] Office of Dietary Supplements Vitamin E. https://ods.od.nih.gov/factsheets/VitaminE-HealthProfessional/.

[18] Anghel L., Baroiu L., Beznea A., Grigore G. (2019). The Therapeutic Relevance of Vitamin E. Rev. Chim..

[19] USDA FoodData Central. https://fdc.nal.usda.gov/.

[20] Csapó J., Albert C., Prokisch J. (2017). The Role of Vitamins in the Diet of the Elderly II. Water-Soluble Vitamins. Acta Univ. Sapientiae Aliment..

[21] Niki E., Abe K. (2019). Vitamin E: Structure, Properties and Functions. Food Chemistry, Function and Analysis.

[22] Colomé C., Artuch R., Vilaseca M.-A., Sierra C., Brandi N., Lambruschini N., Cambra F.J., Campistol J. (2003). Lipophilic Antioxidants in Patients with Phenylketonuria123. Am. J. Clin. Nutr..

[23] Gassió R., Artuch R., Vilaseca M.A., Fusté E., Colome R., Campistol J. (2008). Cognitive Functions and the Antioxidant System in Phenylketonuric Patients. Neuropsychology.

[24] Artuch R., Colomé C., Sierra C., Brandi N., Lambruschini N., Campistol J., Ugarte D., Vilaseca M.A. (2004). A Longitudinal Study of Antioxidant Status in Phenylketonuric Patients. Clin. Biochem..

[25] Artuch R., Colomé C., Vilaseca M.A., Sierra C., Cambra F.J., Lambruschini N., Campistol J. (2001). Plasma Phenylalanine Is Associated with Decreased Serum Ubiquinone-10 Concentrations in Phenylketonuria. J. Inherit. Metab. Dis..

[26] Ribas G.S., Sitta A., Wajner M., Vargas C.R. (2011). Oxidative Stress in Phenylketonuria: What Is the Evidence?. Cell. Mol. Neurobiol..

[27] Kose E., Arslan N. (2019). Vitamin/Mineral and Micronutrient Status in Patients with Classical Phenylketonuria. Clin. Nutr..

[28] Schulpis K.H., Tsakiris S., Karikas G.A., Moukas M., Behrakis P. (2003). Effect of Diet on Plasma Total Antioxidant Status in Phenylketonuric Patients. Eur. J. Clin. Nutr..

[29] Mikoluc B., Motkowski R., Karpinska J., Amilkiewicz J., Didycz B., Gizewska M., Lange A., Milanowski A., Nowacka M., Sands D. (2012). Impact of Lipophilic Antioxidants and Level of Antibodies against Oxidized Low-Density Lipoprotein in Polish Children with Phenylketonuria. Antioxid. Redox Signal..

[30] Office of Dietary Supplements Vitamin D. https://ods.od.nih.gov/factsheets/VitaminD-HealthProfessional/.

[31] Ross A.C., Taylor C.L., Yaktine A.L., Del Valle H.B., Institute of Medicine (US) Committee to Review (2011). Dietary Reference Intakes for Vitamin D and Calcium. Dietary Reference Intakes for Calcium and Vitamin D.

[32] Goltzman D. (2018). Functions of Vitamin D in Bone. Histochem. Cell Biol..

[33] Mutt S.J., Karhu T., Lehtonen S., Lehenkari P., Carlberg C., Saarnio J., Sebert S., Hyppönen E., Järvelin M.-R., Herzig K.-H. (2012). Inhibition of Cytokine Secretion from Adipocytes by 1,25-Dihydroxyvitamin D_3_ via the NF-κB Pathway. FASEB J..

[34] Mutt S.J., Raza G.S., Mäkinen M.J., Keinänen-Kiukaanniemi S., Järvelin M.-R., Herzig K.-H. (2020). Vitamin D Deficiency Induces Insulin Resistance and Re-Supplementation Attenuates Hepatic Glucose Output via the PI3K-AKT-FOXO1 Mediated Pathway. Mol. Nutr. Food Res..

[35] Arslan S., Akdevelioğlu Y. (2018). The Relationship Between Female Reproductive Functions and Vitamin D. J. Am. Coll. Nutr..

[36] Medrano M., Carrillo-Cruz E., Montero I., Perez-Simon J.A. (2018). Vitamin D: Effect on Haematopoiesis and Immune System and Clinical Applications. Int. J. Mol. Sci..

[37] Lumme J., Morin-Papunen L., Pesonen P., Sebert S., Hyppönen E., Järvelin M.-R., Herzig K.-H., Ojaniemi M., Niinimäki M. (2023). Vitamin D Status in Women with a History of Infertility and Decreased Fecundability: A Population-Based Study. Nutrients.

[38] de La Puente-Yagüe M., Cuadrado-Cenzual M.A., Ciudad-Cabañas M.J., Hernández-Cabria M., Collado-Yurrita L. (2018). Vitamin D: And Its Role in Breast Cancer. Kaohsiung J. Med. Sci..

[39] Trump D.L., Aragon-Ching J.B. (2018). Vitamin D in Prostate Cancer. Asian J. Androl..

[40] Casseb G.A.S., Kaster M.P., Rodrigues A.L.S. (2019). Potential Role of Vitamin D for the Management of Depression and Anxiety. CNS Drugs.

[41] Leiva C., Bravo P., Arias C., Cabello J.F., Leal-Witt M.J., Salazar F., Cornejo V. (2021). 25 Hydroxy Vitamin D Level, Bone Health, Vitamin D and Calcium Intake in Chilean Patients with Phenylketonuria and Hyperphenylalaninemias. J. Inborn Errors Metab. Screen..

[42] Bokayeva K., Jamka M., Walkowiak D., Duś-Żuchowska M., Herzig K.-H., Walkowiak J. (2024). Vitamin Status in Patients with Phenylketonuria: A Systematic Review and Meta-Analysis. Int. J. Mol. Sci..

[43] Silva M.F.S., Leal-Witt M.J., Hamilton V., Cornejo V., Silva M.F.S., Leal-Witt M.J., Hamilton V., Cornejo V. (2023). Vitamin D and Inborn Errors of Metabolism.

[44] Rojas-Agurto E., Leal-Witt M.J., Arias C., Cabello J.F., Bunout D., Cornejo V. (2023). Muscle and Bone Health in Young Chilean Adults with Phenylketonuria and Different Degrees of Compliance with the Phenylalanine Restricted Diet. Nutrients.

[45] Hochuli M., Bollhalder S., Thierer C., Refardt J., Gerber P., Baumgartner M.R. (2017). Effects of Inadequate Amino Acid Mixture Intake on Nutrient Supply of Adult Patients with Phenylketonuria. Ann. Nutr. Metab..

[46] Anton-Păduraru D.-T., Trofin F., Chis A., Sur L.M., Streangă V., Mîndru D.E., Dorneanu O.S., Păduraru D., Nastase E.V., Vulturar R. (2025). Current Insights into Nutritional Management of Phenylketonuria: An Update for Children and Adolescents. Children.

[47] Mancilla V.J., Mann A.E., Zhang Y., Allen M.S. (2021). The Adult Phenylketonuria (PKU) Gut Microbiome. Microorganisms.

[48] Ostrowska M., Nowosad K., Mikoluc B., Szczerba H., Komon-Janczara E. (2024). Changes in the Gut and Oral Microbiome in Children with Phenylketonuria in the Context of Dietary Restrictions-A Preliminary Study. Nutrients.

[49] Pinheiro de Oliveira F., Mendes R.H., Dobbler P.T., Mai V., Pylro V.S., Waugh S.G., Vairo F., Refosco L.F., Roesch L.F.W., Schwartz I.V.D. (2016). Phenylketonuria and Gut Microbiota: A Controlled Study Based on Next-Generation Sequencing. PLoS ONE.

[50] Montanari C., Ceccarani C., Corsello A., Zuvadelli J., Ottaviano E., Dei Cas M., Banderali G., Zuccotti G., Borghi E., Verduci E. (2022). Glycomacropeptide Safety and Its Effect on Gut Microbiota in Patients with Phenylketonuria: A Pilot Study. Nutrients.

[51] Ramos Meyers G., Samouda H., Bohn T. (2022). Short Chain Fatty Acid Metabolism in Relation to Gut Microbiota and Genetic Variability. Nutrients.

[52] Di Profio E., Magenes V.C., Fiore G., Agostinelli M., La Mendola A., Acunzo M., Francavilla R., Indrio F., Bosetti A., D’Auria E. (2022). Special Diets in Infants and Children and Impact on Gut Microbioma. Nutrients.

[53] von Elm E., Altman D.G., Egger M., Pocock S.J., Gøtzsche P.C., Vandenbroucke J.P., STROBE Initiative (2008). The Strengthening the Reporting of Observational Studies in Epidemiology (STROBE) Statement: Guidelines for Reporting Observational Studies. J. Clin. Epidemiol..

[54] Nowak J.K., Walkowiak J. (2023). Study Designs in Medical Research and Their Key Characteristics. J. Med. Sci..

[55] Malone H.E., Nicholl H., Coyne I. (2016). Fundamentals of Estimating Sample Size. Nurse Res..

[56] Cole T.J., Lobstein T. (2012). Extended International (IOTF) Body Mass Index Cut-Offs for Thinness, Overweight and Obesity. Pediatr. Obes..

[57] Sapiejka E., Krzyżanowska-Jankowska P., Wenska-Chyży E., Szczepanik M., Walkowiak D., Cofta S., Pogorzelski A., Skorupa W., Walkowiak J. (2018). Vitamin E Status and Its Determinants in Patients with Cystic Fibrosis. Adv. Med. Sci..

[58] Sapiejka E., Krzyżanowska P., Walkowiak D., Wenska-Chyży E., Szczepanik M., Cofta S., Pogorzelski A., Skorupa W., Walkowiak J. (2017). Vitamin A Status and Its Determinants in Patients with Cystic Fibrosis. Acta Sci. Pol. Technol. Aliment..

[59] Raizman J.E., Cohen A.H., Teodoro-Morrison T., Wan B., Khun-Chen M., Wilkenson C., Bevilaqua V., Adeli K. (2014). Pediatric Reference Value Distributions for Vitamins A and E in the CALIPER Cohort and Establishment of Age-Stratified Reference Intervals. Clin. Biochem..

[60] Johnson-Davis K.L., Moore S.J., Owen W.E., Cutler J.M., Frank E.L. (2009). A Rapid HPLC Method Used to Establish Pediatric Reference Intervals for Vitamins A and E. Clin. Chim. Acta.

[61] Rusińska A., Płudowski P., Walczak M., Borszewska-Kornacka M.K., Bossowski A., Chlebna-Sokół D., Czech-Kowalska J., Dobrzańska A., Franek E., Helwich E. (2018). Vitamin D Supplementation Guidelines for General Population and Groups at Risk of Vitamin D Deficiency in Poland—Recommendations of the Polish Society of Pediatric Endocrinology and Diabetes and the Expert Panel with Participation of National Specialist Consultants and Representatives of Scientific Societies—2018 Update. Front. Endocrinol..

[62] Le N.K., Kesayan T., Chang J.Y., Rose D.Z. (2020). Cryptogenic Intracranial Hemorrhagic Strokes Associated with Hypervitaminosis E and Acutely Elevated α-Tocopherol Levels. J. Stroke Cerebrovasc. Dis..

[63] 001529: Carotene, β. https://www.labcorp.com/tests/001529/carotene-b.

[64] FCATB—Overview: Carotene, Beta. https://www.mayocliniclabs.com/test-catalog/overview/75927#Clinical-and-Interpretive.

[65] Sawicka-Gutaj N., Gruszczyński D., Guzik P., Mostowska A., Walkowiak J. (2022). Publication Ethics of Human Studies in the Light of the Declaration of Helsinki—A Mini-Review. J. Med. Sci..

[66] Posit Team (2024). RStudio: Integrated Development Environment for R.

[67] Page M.J., McKenzie J.E., Bossuyt P.M., Boutron I., Hoffmann T.C., Mulrow C.D., Shamseer L., Tetzlaff J.M., Akl E.A., Brennan S.E. (2021). The PRISMA 2020 Statement: An Updated Guideline for Reporting Systematic Reviews. BMJ.

[68] Higgins J.P.T., Thomas J., Chandler J., Cumpston M., Li T., Page M.J., Welch V.A. Cochrane Handbook for Systematic Reviews of Interventions Version 6.5 (Updated August 2024). Cochrane, 2024. https://www.cochrane.org/authors/handbooks-and-manuals/handbook/current.

[69] Bokayeva K., Jamka M., Walkowiak D., Herzig K.-H., Walkowiak J. Fat-Soluble Vitamins Comparison in Adherent vs. Non-Adherent and Regular Formula Intake vs. Irregular Formula Intake Individuals with PKU: Systematic Review and Meta-Analysis. https://www.crd.york.ac.uk/PROSPERO/view/CRD420251128538.

[70] Wells G.A., Shea B., O’Connell D., Peterson J., Welch V., Losos M., Tugwell P. (2025). The Newcastle-Ottawa Scale (NOS) for Assessing the Quality of Non-Randomized Studies in Meta-Analyses.

[71] Modesti P.A., Reboldi G., Cappuccio F.P., Agyemang C., Remuzzi G., Rapi S., Perruolo E., Parati G. (2016). ESH Working Group on CV Risk in Low Resource Settings. Panethnic Differences in Blood Pressure in Europe: A Systematic Review and Meta-Analysis. PLoS ONE.

[72] Puhan M.A., Schünemann H.J., Murad M.H., Li T., Brignardello-Petersen R., Singh J.A., Kessels A.G., Guyatt G.H. (2014). A GRADE Working Group Approach for Rating the Quality of Treatment Effect Estimates from Network Meta-Analysis. BMJ.

[73] Crujeiras V., Aldámiz-Echevarría L., Dalmau J., Vitoria I., Andrade F., Roca I., Leis R., Fermandez-Marmiesse A., Couce M.L. (2015). Micronutrient in Hyperphenylalaninemia. Data Brief.

[74] Kol B.A., Papur Ö.Ş., Kulu B., Arslan N. (2025). Retrospective Evaluation of Diet Compliance on Plasma Amino Acid and Vitamin Levels in Patients with Phenylketonuria. JBACHS.

[75] Vockley J., Andersson H.C., Antshel K.M., Braverman N.E., Burton B.K., Frazier D.M., Mitchell J., Smith W.E., Thompson B.H., Berry S.A. (2014). Phenylalanine Hydroxylase Deficiency: Diagnosis and Management Guideline. Genet. Med..

[76] Palacios C., Gonzalez L. (2014). Is Vitamin D Deficiency a Major Global Public Health Problem?. J. Steroid Biochem. Mol. Biol..

[77] Holick M.F. (2011). Vitamin D: A D-Lightful Solution for Health. J. Investig. Med..

[78] Robertson L.V., McStravick N., Ripley S., Weetch E., Donald S., Adam S., Micciche A., Boocock S., MacDonald A. (2013). Body Mass Index in Adult Patients with Diet-Treated Phenylketonuria. J. Hum. Nutr. Diet..

[79] Gokmen Ozel H., Ahring K., Bélanger-Quintana A., Dokoupil K., Lammardo A.M., Robert M., Rocha J.C., Almeida M.F., van Rijn M., MacDonald A. (2014). Overweight and Obesity in PKU: The Results from 8 Centres in Europe and Turkey. Mol. Genet. Metab. Rep..

[80] Barta A.G., Becsei D., Kiss E., Sumánszki C., Simonová E., Reismann P. (2021). The Impact of Phenylketonuria on Body Composition in Adults. Ann. Nutr. Metab..

[81] Tankeu A.T., Pavlidou D.C., Superti-Furga A., Gariani K., Tran C. (2023). Overweight and Obesity in Adult Patients with Phenylketonuria: A Systematic Review. Orphanet J. Rare Dis..

[82] Pilz S., März W., Cashman K.D., Kiely M.E., Whiting S.J., Holick M.F., Grant W.B., Pludowski P., Hiligsmann M., Trummer C. (2018). Rationale and Plan for Vitamin D Food Fortification: A Review and Guidance Paper. Front. Endocrinol..

[83] Schmid A., Walther B. (2013). Natural Vitamin D Content in Animal Products. Adv. Nutr..

[84] Holick M.F. (2007). Vitamin D Deficiency. N. Engl. J. Med..

[85] Venegas E., Langeveld S., Ahring K., Benitez R., Desloovere A., Dios E., Gómez E., Hermida A., Marsaux C., Verloo P. (2024). Nutrient Status and Intakes of Adults with Phenylketonuria. Nutrients.

[86] Geiger K.E., Koeller D.M., Harding C.O., Huntington K.L., Gillingham M.B. (2016). Normal Vitamin D Levels and Bone Mineral Density among Children with Inborn Errors of Metabolism Consuming Medical Food–Based Diets. Nutr. Res..

[87] Hakeem M.K., El-Konaissi I., Alzohily B., Gariballa S., Yasin J., Shah I. (2024). Evaluating a Novel Method for Vitamin A Analysis in an Observational Study of the UAE’s Obese Population. Sci. Rep..

[88] Vitamin A Toxicity—Nutritional Disorders. https://www.msdmanuals.com/professional/nutritional-disorders/vitamin-deficiency-dependency-and-toxicity/vitamin-a-toxicity.

[89] Menezes M.S.S., Almeida C.M.M. (2024). Structural, Functional, Nutritional and Clinical Aspects of Vitamin A: A Review. PharmaNutrition.

[90] Kiyose C. (2021). Absorption, Transportation, and Distribution of Vitamin E Homologs. Free Radic. Biol. Med..

[91] Beta Carotene (Oral Route)—Side Effects & Dosage. https://www.mayoclinic.org/drugs-supplements/beta-carotene-oral-route/description/drg-20066795.

[92] Nagao A., Kotake-Nara E., Hase M. (2013). Effects of Fats and Oils on the Bioaccessibility of Carotenoids and Vitamin E in Vegetables. Biosci. Biotechnol. Biochem..

[93] Hörster F., Schwab M.A., Sauer S.W., Pietz J., Hoffmann G.F., Okun J.G., Kölker S., Kins S. (2006). Phenylalanine Reduces Synaptic Density in Mixed Cortical Cultures from Mice. Pediatr. Res..

[94] Sirtori L.R., Dutra-Filho C.S., Fitarelli D., Sitta A., Haeser A., Barschak A.G., Wajner M., Coelho D.M., Llesuy S., Belló-Klein A. (2005). Oxidative Stress in Patients with Phenylketonuria. Biochim. Biophys. Acta.

[95] Fernandes C.G., Leipnitz G., Seminotti B., Amaral A.U., Zanatta Â., Vargas C.R., Dutra Filho C.S., Wajner M. (2009). Experimental Evidence That Phenylalanine Provokes Oxidative Stress in Hippocampus and Cerebral Cortex of Developing Rats. Cell. Mol. Neurobiol..

