# Supplementary figures and images for: Vitamins D, A and E, and Beta-Carotene in Adherent and Non-Adherent Individuals with Phenylketonuria: Cross-Sectional Study, Systematic Review and Meta-Analysis
Source: Nutrients. 2025 Dec 16;17(24):3932. doi: 10.3390/nu17243932 (PMC12735712; doi:10.3390/nu17243932)

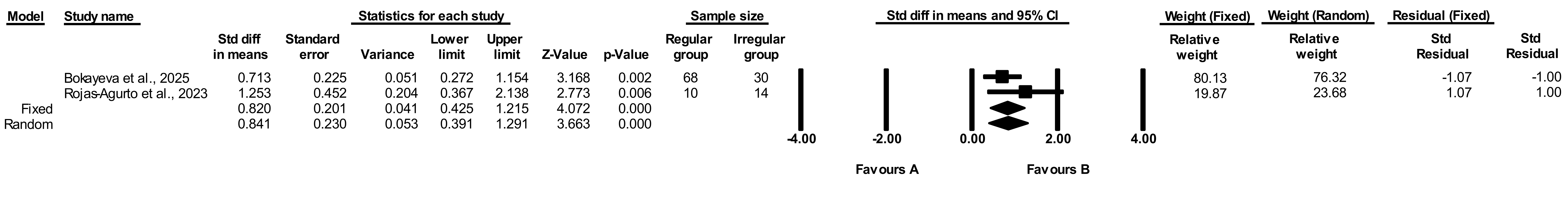

Supplement: Supplementary file 1 [file nutrients-17-03932-s001.zip › Figure S8. Vitamin D without hrob (regular vs irregular).png]

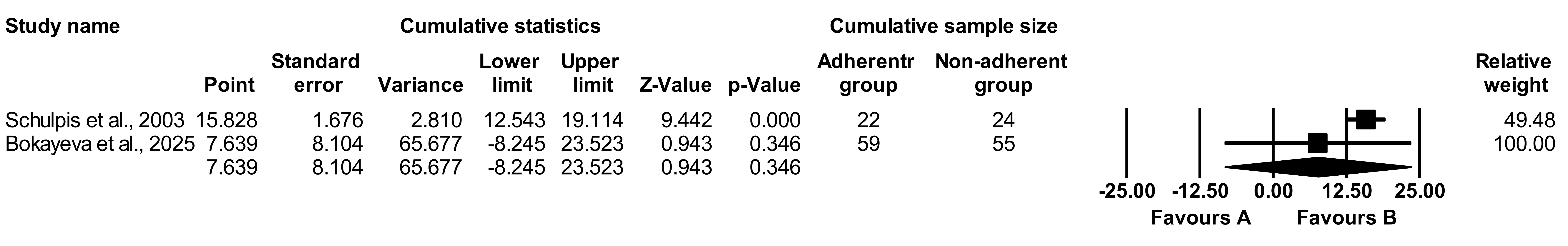

Supplement: Supplementary file 1 [file nutrients-17-03932-s001.zip › Figure S9. Vitamin E cumulative analysis.png]

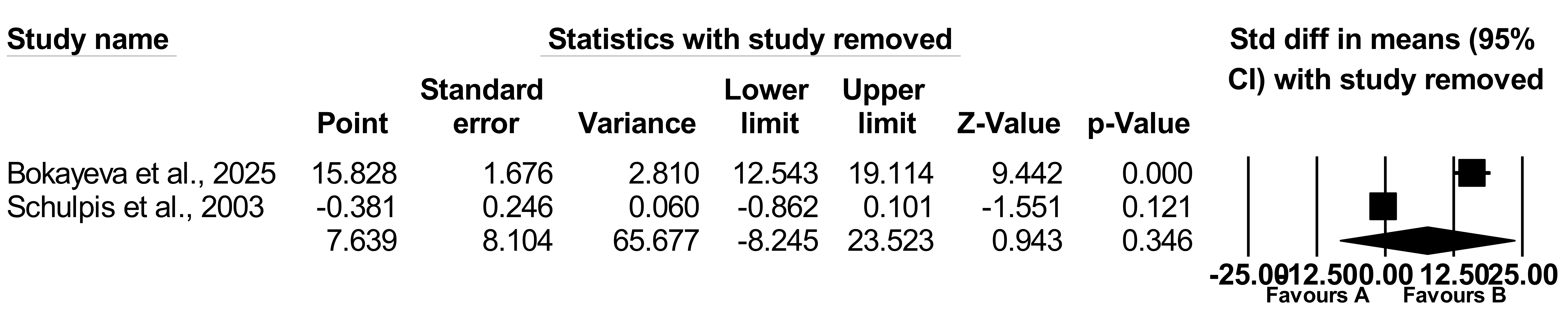

Supplement: Supplementary file 1 [file nutrients-17-03932-s001.zip › Figure S10. Vitamin E sensitivity analysis.png]

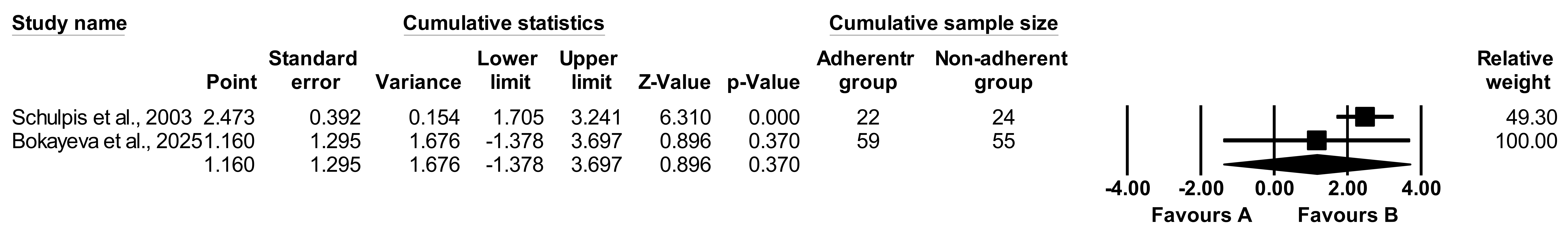

Supplement: Supplementary file 1 [file nutrients-17-03932-s001.zip › Figure S11. Beta-carotene cumulative analysis.png]

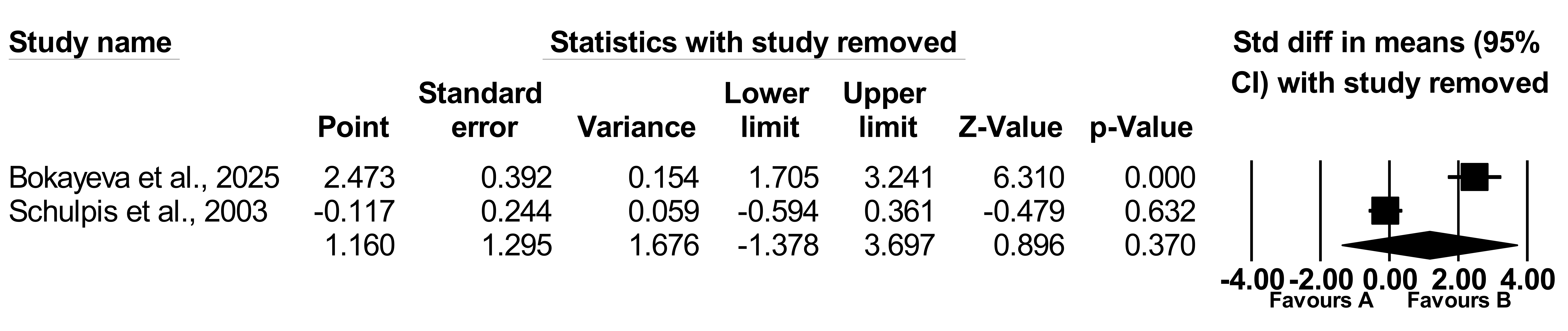

Supplement: Supplementary file 1 [file nutrients-17-03932-s001.zip › Figure S12. Beta-carotene sensitivity analysis.png]

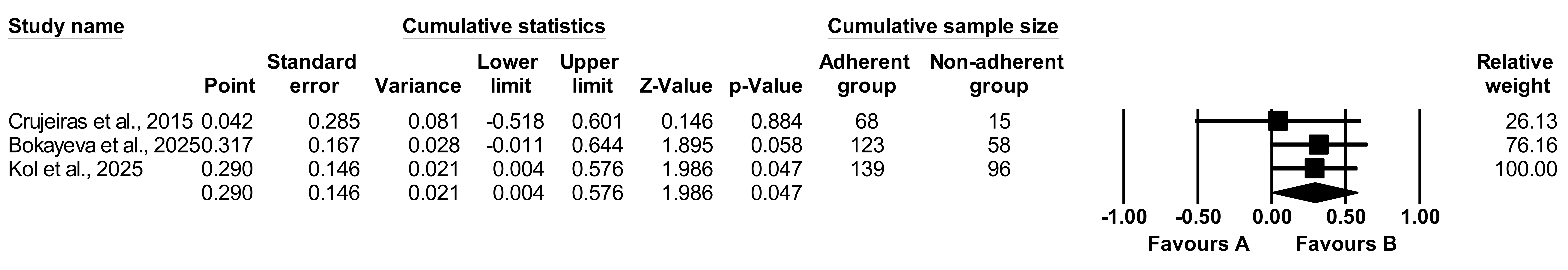

Supplement: Supplementary file 1 [file nutrients-17-03932-s001.zip › Figure S1. Vitamin D cumulative analysis (adherent vs non-adherent).png]

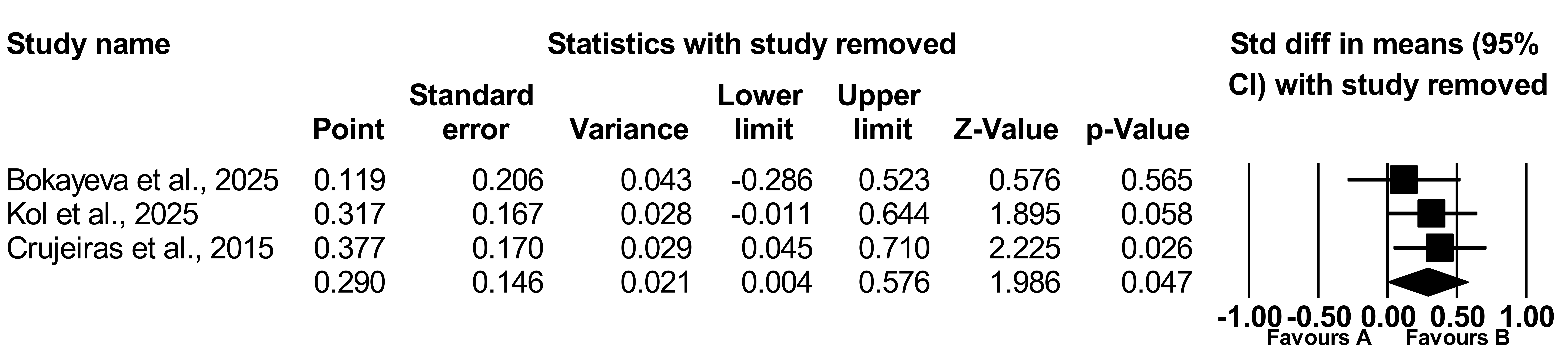

Supplement: Supplementary file 1 [file nutrients-17-03932-s001.zip › Figure S2. Vitamin D sensitivity analysis (adherent vs non-adherent).png]

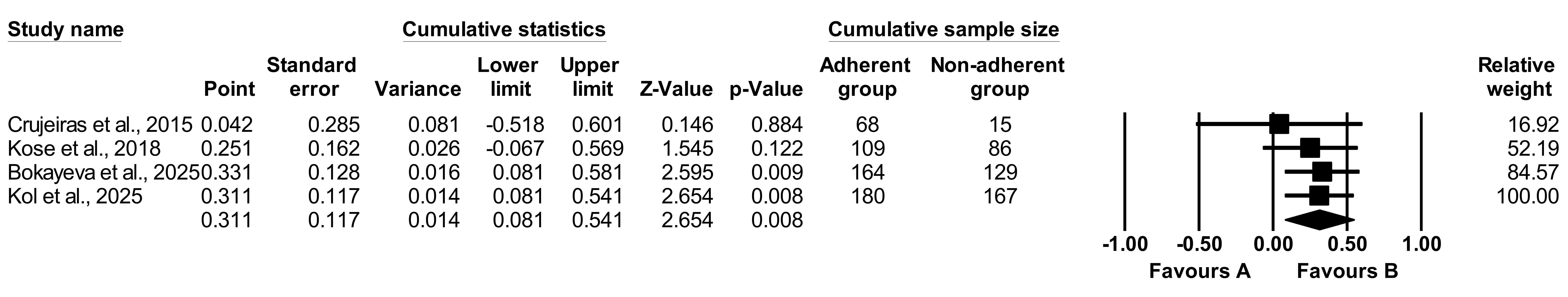

Supplement: Supplementary file 1 [file nutrients-17-03932-s001.zip › Figure S3. Vitamin D Expanded cumulative analysis (adherent vs non-adherent).png]

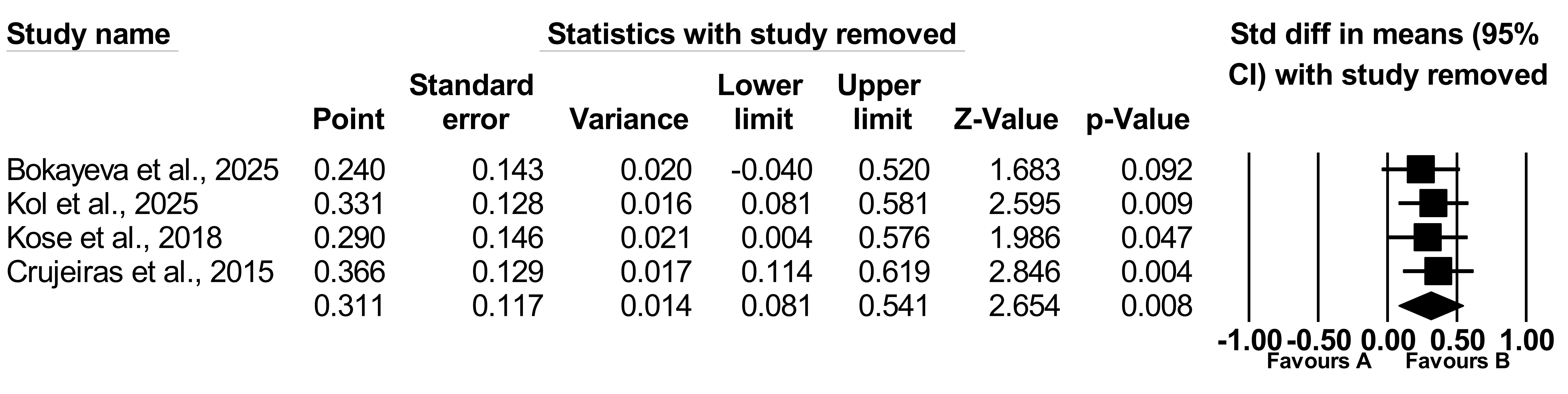

Supplement: Supplementary file 1 [file nutrients-17-03932-s001.zip › Figure S4. Vitamin D Expanded sensitivity analysis (adherent vs non-adherent).png]

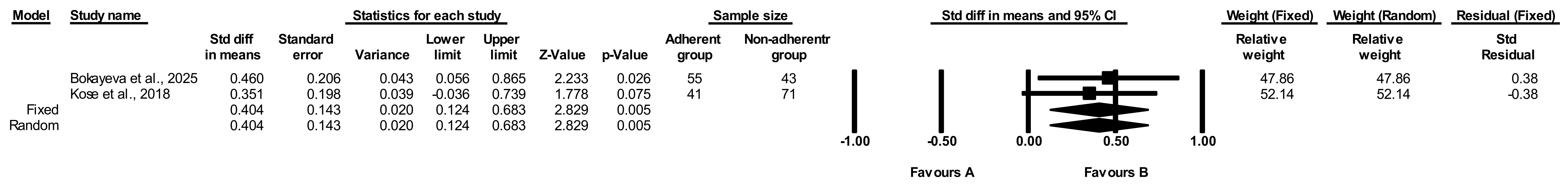

Supplement: Supplementary file 1 [file nutrients-17-03932-s001.zip › Figure S5. Vitamin D Expanded without hrob (adherent vs non-adherent).png]

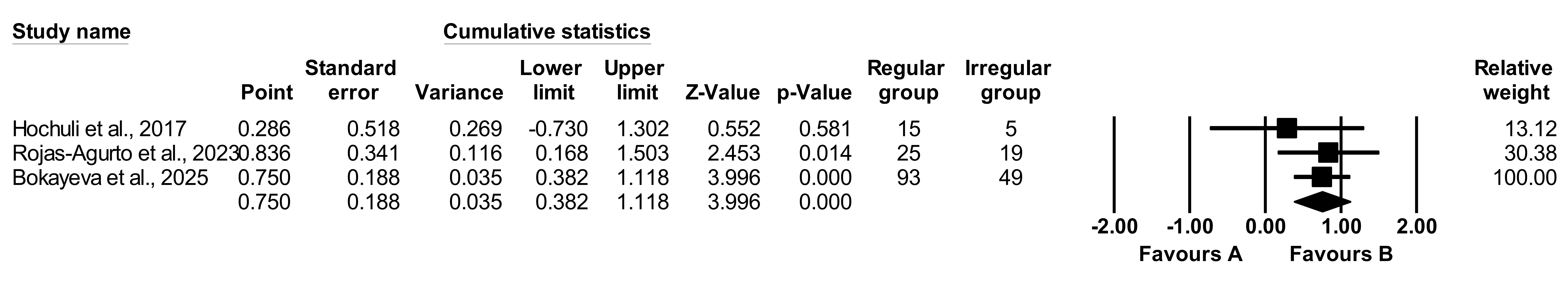

Supplement: Supplementary file 1 [file nutrients-17-03932-s001.zip › Figure S6. Vitamin D cumulative analysis (regular vs irregular).png]

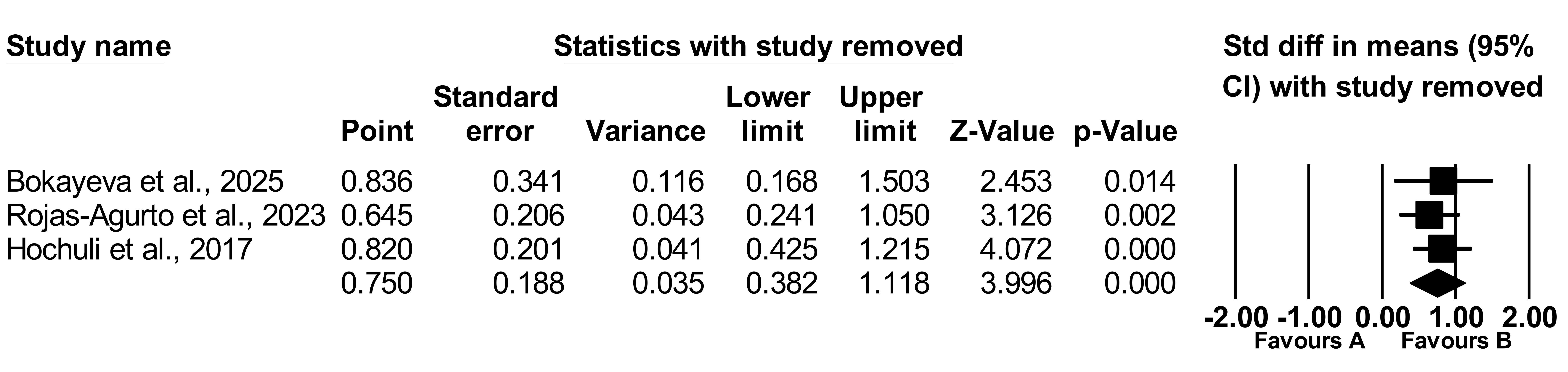

Supplement: Supplementary file 1 [file nutrients-17-03932-s001.zip › Figure S7. Vitamin D sensitivity analysis (regular vs irregular).png]
